# Rapid assembly of multilayer microfluidic structures via 3D-printed transfer molding and bonding

**DOI:** 10.1038/micronano.2016.63

**Published:** 2016-11-21

**Authors:** Casey C. Glick, Mitchell T. Srimongkol, Aaron J. Schwartz, William S. Zhuang, Joseph C. Lin, Roseanne H. Warren, Dennis R. Tekell, Panitan A. Satamalee, Liwei Lin

**Affiliations:** 1 Department of Physics, University of California, Berkeley, CA 94720, USA; 2 Department of Mechanical Engineering, University of California, Berkeley, CA 94720, USA; 3 Department of Mechanical Engineering, University of Utah, Salt Lake City, UT 84112, USA

**Keywords:** 3D printing, microfluidics, PDMS

## Abstract

A critical feature of state-of-the-art microfluidic technologies is the ability to fabricate multilayer structures without relying on the expensive equipment and facilities required by soft lithography-defined processes. Here, three-dimensional (3D) printed polymer molds are used to construct multilayer poly(dimethylsiloxane) (PDMS) devices by employing unique molding, bonding, alignment, and rapid assembly processes. Specifically, a novel single-layer, two-sided molding method is developed to realize two channel levels, non-planar membranes/valves, vertical interconnects (vias) between channel levels, and integrated inlet/outlet ports for fast linkages to external fluidic systems. As a demonstration, a single-layer membrane microvalve is constructed and tested by applying various gate pressures under parametric variation of source pressure, illustrating a high degree of flow rate control. In addition, multilayer structures are fabricated through an intralayer bonding procedure that uses custom 3D-printed stamps to selectively apply uncured liquid PDMS adhesive only to bonding interfaces without clogging fluidic channels. Using integrated alignment marks to accurately position both stamps and individual layers, this technique is demonstrated by rapidly assembling a six-layer microfluidic device. By combining the versatility of 3D printing while retaining the favorable mechanical and biological properties of PDMS, this work can potentially open up a new class of manufacturing techniques for multilayer microfluidic systems.

## Introduction

Microfluidic devices for manipulating fluids have rapidly advanced since the 1980s because of their unique ability to fabricate low-cost, high-throughput platforms, particularly for chemical and biological research and lab-on-a-chip technologies^1,2^. The most far-reaching breakthrough in microfluidics has been the development of soft lithography: using rigid micromachined molds to pattern elastomeric polymers^3^. Among polymeric materials, poly(dimethylsiloxane) (PDMS) is commonly used because of its numerous favorable properties, including its ease in manufacturing, reasonable cost, strength, transparency, and especially biocompatibility^4^.

Traditional methods for fabricating microfluidic devices involve using photolithography to construct micromolds with very fine features; however, this process can be long and costly. The increasing demand for microfluidics is particularly high for multilayered devices featuring more sophisticated structures and components (including valves, pumps, and other active control mechanisms). For example, soft lithography through micromachining processes is generally restricted to monolithic rectilinear features^5^, although rounded and fully circular channels are common in large-scale fluidic systems.

One method of increasing geometric complexity is 'multilevel soft lithography'^6^, in which the channels are non-planar and/or rounded^7^. Although rounded channels are beneficial for some microfluidic applications, few groups have developed appropriate fabrication techniques (Supplementary Material S3.4)^8–10^ because multilevel soft lithography has historically required multiple photolithography steps^11^. Although “grayscale lithography”—whereby resists are exposed to non-binary shades of gray—can potentially generate rounded microfluidic channels^12–14^, the process still requires multiple exposures to obtain larger aspect ratios^15,16^. Furthermore, although multilayer PDMS-manufacturing techniques have been demonstrated by several groups^17,18^, these are even more time-consuming and labor-intensive, requiring multiple lithography steps and precision alignment, issues that are only partially addressed by dedicated PDMS-alignment tools^19^.

Three-dimensional (3D) printing offers a unique route for building multilevel and multilayer microfluidic devices directly, or indirectly via molding processes. For example, various groups have used 3D printers to fabricate simple microfluidic devices with truly 3D geometries, including microfluidic devices without moving elements, such as resistors^20^ and modular components^21^, as well as those with movable components, such as capacitors, diodes, and transistors^22^. Currently, the field of 3D-printed microfluidics is limited by the following: (1) the available resolution of the printer^20^; (2) surface roughness^23,24^; and (3) material types^25,26^; however, 3D printing technologies are expected to rapidly advance and address these matters in the coming years. For further details on current 3D printer capabilities, including printer resolution and surface roughness, see reviews in Refs. 27–32.

Although direct 3D printing is a rapid process for prototyping, for making multiple copies of microfluidic devices, 3D-printed transfer molding (PTM), in which polymer is poured into a 3D-printed mold, remains faster, cheaper, and more reliable. First pioneered by McDonald *et al.*^33^ using fused deposition modeling techniques, the technique has since been used with stereolithography^34^ and multijet printing^23^ as well as with wax printers^35^ and office-quality laser printers^36,37^. Although PTM does not allow the geometric flexibility of fully 3D-printed microfluidics, it possesses the following three notable advantages: (i) each mold can be used for multiple microfluidic devices, reducing 3D printing times and costs, (ii) many 3D printers exhibit lower resolution for features requiring support materials, and (iii) the process is compatible with conventional microfluidic fabrication materials, most notably PDMS^34,35,37–39^. Because PDMS can be used to transfer patterns with high fidelity, the resolution and surface finish of the mold define the resulting quality of the resulting PDMS structure^38,40^, provided the mold features reasonable aspect ratios^23^.

In addition to patterning features externally, PTM has also been used to fabricate internal features, reducing certain monolithic constraints. Hwang *et al.*^41,42^ have developed printed molds that are enveloped by PDMS and then withdrawn after curing, relying on the flexibility of PDMS to remove the components. Although similar to fugitive ink processes^43–45^, fugitive ink molding has fewer geometric constraints but requires a printing step for every final device, whereas solid internal structures must be designed for withdrawal but can be reused^23,41,42^. Chan *et al.*^46^ have fabricated molds with overhangs in a basket weave pattern, which can be used to generate microfluidic vias and valving in a single step, repairing demolding damage by thermally healing the PDMS, with the restriction that the vias be designed in parallel.

PTM is also increasingly integrated with other 3D printing processes to reduce the challenges of multicomponent assembly and to interface microfluidic devices with external systems^29^. Although some PTM devices can interface directly, whether, by punching holes (in a manner, similar to standard soft lithography processes)^36^, molding open wells^33,47^, and adding connectors during the curing process^34,37,48^, other PTM devices can interface indirectly by attaching to 3D-printed components that do have the desired interfaces^39^. Similar to soft lithography, PTM devices are sealed with glass or other 3D-printed components to provide enclosed channels after molding (including, through plasma bonding^33^, mechanical pressure^39^, or tape^39^).

This work advances 3D PTM techniques from single-sided microfluidics^33,34,37^ to multilayer microfluidic manufacturing, using the ease of 3D printing to create multiple molds with alignment structures to shape multiple layers of PDMS structures and quickly assemble them in the final step. First, we discuss details specific to the ProJet™ 3000 3D printer, including resolution and surface treatments. Next, we examine the single-step double-sided molding method used to create PDMS components with complex geometries including vias, thin membranes, and rounded channels that are difficult to achieve using standard soft lithography, as well as integrated input/output marks that do not require positioning external components during PDMS curing. Finally, we demonstrate rapid assembly of multilayer microfluidic devices including integrated alignment marks, which enable tactile—as opposed to optical—alignment of layers to within the resolution of the 3D printer (including PDMS–PDMS for multilayer assembly and mold–PDMS for interfacing with other 3D-printed objects), and bonding techniques, including a specialized variant of adhesive bonding techniques introduced by Satyanarayana *et al.*^49^. Using custom 3D printing to selectively apply a thin layer of liquid PDMS adhesive to non-channel areas, layers were successfully bonded without the adhesive clogging narrow channels, obviating problems associated with mold surface roughness.

## Materials and methods

### 3D-printed molds

Microfluidic components were designed and converted from a positive to a negative mold shape using the computer-aided-design program SolidWorks. 3D printing of molds was achieved using a ProJet™ 3000 3D printer (3D Systems, Rock Hill, SC, USA)^50^. During printing, the ProJet™ 3000 alternately deposited 3D Systems proprietary structural epoxy (VisiJet®EX200 plastic material^51,52^) and sacrificial wax support material (VisiJet®S100 hydroxylated wax^53^) in 0.35 μm layers. The wax was used as a temporary support for cavities and overhangs and was removed during post processing. The printer was capable of resolving extruded features as small as 50 μm and intruded features as small as 100 μm. For more information on chemical and material properties, see Supplementary Material S1.

### Mold post processing

Following printing, the molds were cleaned to remove the sacrificial wax. First, the molds were baked in a VWR 1330 FM oven (VWR, Radnor, PA, USA) at 75 °C for 45 min to melt the sacrificial wax. The molds were then washed in a sequence of three cleaning baths for 10 min in each bath to remove leftover wax: warm Bayes mineral oil, Ajax dish detergent in water, and potable water. The baths were heated to 75 °C to ensure that the wax did not solidify and were placed on a hotplate with a magnetic stir bar to enhance the removal of wax, oil, and soap, respectively. The molds were then dried by baking at 80 °C for 60 min. After cleaning and drying, the 3D-printed molds were treated with an fluorinated silane anti-adhesive agent, trichloro(1H,1H,2H,2H-perfluorooctyl)silane gas (PFOTS, Sigma-Aldrich, St Louis, MO, USA) to make the surface hydrophobic and to facilitate the rapid removal of PDMS. Next, the molds and a 1.5-cm Outer Diameter (OD) glass vial containing ≈0.3 ml PFOTS agent were placed in a vacuum desiccator (10^−4^ torr) for 30 min for the vapor treatment. Shorter treatment times resulted in PDMS bonding to the mold, and longer treatment times caused a build-up of PFOTS, which inhibited complete curing of the PDMS near the surface (Supplementary Material S2.3)^54,55^.

### PDMS molding

The 3D-printed molds were placed on a foil-wrapped 3D-printed molding tray (Supplementary Material S2.1) shaped to substantially reduce PDMS waste. PDMS (Sylgard 184 Elastomer Kit, Dow Corning, Midlind, MI, USA) was prepared using the standard 10:1 base:curing agent ratio. The PDMS mixture was degassed in a vacuum chamber to 10^−4^ torr (Supplementary Material S7) for 10 min and poured on the 3D-printed molds. The filled molds were then returned to the vacuum chamber for 30 min to degas and increase PDMS conformity. For double-sided molding processes, this degassing also serves to load uncured PDMS between the upper and lower molds. Following degassing, the molds were baked in an 80 °C oven for 50 min. The PDMS microfluidic components were removed from the molds by first cutting away excess PDMS using the edge of the mold as a guide and then by manually peeling the PDMS from the mold. This step was performed carefully to avoid damaging the higher aspect features; without structural features such as widened bases and fillets, many devices lost at least one input/output port within 5–10 demolding events because of handling error (Supplementary Material S2.2). Provided that no features were broken during the demolding process and PDMS did not permanently bond to the mold, the molds were reused without an additional cleaning process. Approximately every 10–20 moldings, mold hydrophobicity was refreshed by repeating the PFOTS treatment, which was performed when PDMS began adhering excessively to the printed mold (Supplementary Figure S7). For further discussion of molding techniques, see Supplementary Material S2.

## Results

**Figure 1:**
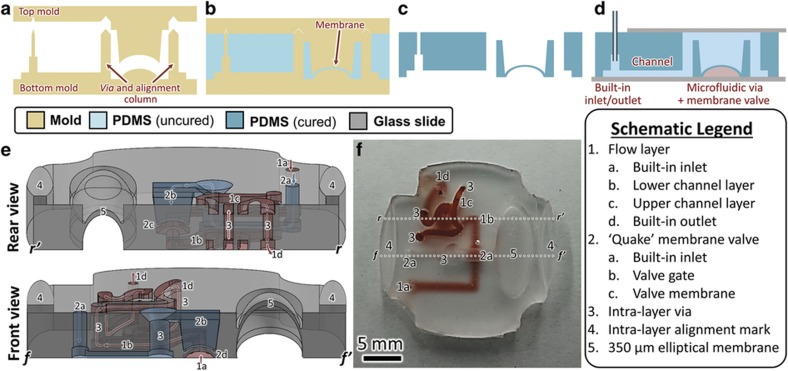
Illustration of fabrication process (top) and technical capabilities (bottom) of 3D-printed transfer molding for double-sided microfluidic devices. Fabrication: (**a**) mold is 3D-printed from a CAD model, treated, fitted using alignment marks, and (**b**) filled with PDMS and cured. Excess PDMS is cut away and the mold is removed. (**c**) The resulting PDMS component contains integrated inlets/outlets, membranes, and vias, and is (**d**) bonded to glass to create a device with enclosed channels of arbitrary cross-section. Technical capabilities: the multilevel microfluidic device shown in cross-section in (**e**) and photographed in (**f**) is fabricated using double-sided molding techniques and exhibits numerous design elements, such as two-layer fluid flow, multiple microfluidic vias, integrated fluid inlets/outlets, an elliptical 350-μm domed membrane, and a “Quake”-style membrane value, as well as alignment marks for use in generating multilayer devices. 3D, three-dimensional; PDMS, poly(dimethylsiloxane).

Figure 1 illustrates the process flow for fabricating multisided PDMS microfluidic devices using 3D-printed molds. The component mold is fabricated via the 3D printing process (Figure 1a), and PDMS is applied (Figure 1c), cured, and released from the mold (Figure 1c) by means of the PDMS molding steps described in Section ‘MATERIALS AND METHODS’. Integrated fluid inlets (diameter 0.55 mm) are easily incorporated into the component through the mold design, which further simplifies fabrication by eliminating the need for an additional hole-punching step. The PDMS component is then bonded to glass to create a complete microfluidic device with enclosed channels (Figure 1d).

**Figure 2:**
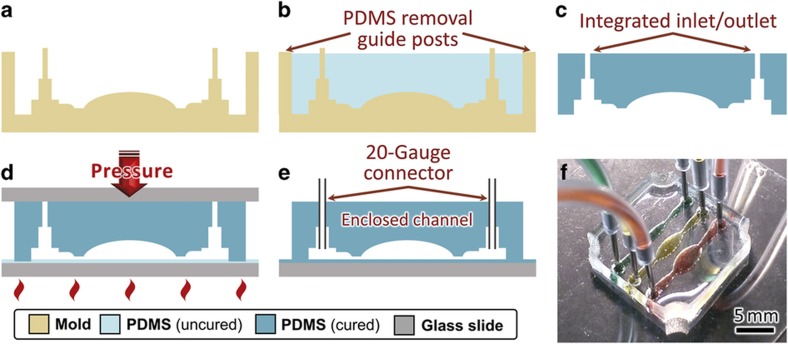
Single-sided fabrication and bonding process flow. (**a**) 3D-printed mold is printed from CAD file, including integrated inlet/outlet ports and guideposts to assist the removal of PDMS. (**b**) Mold is filled with ℓPDMS, degassed, and baked, and (**c**) cured PDMS is demolded. (**d**) Cured PDMS is bonded to glass using the PDMS–glass ℓPDMS spin-bonding technique to compensate for surface roughness. (**e**) Final conceptual image with enclosed channel and 20-gauge connector pins attached. (**f**) Photograph of glass-bonded device with colored fluid. 3D, three-dimensional; PDMS, poly(dimethylsiloxane).

This 3D PTM and bonding technique can be used to fabricate conventional microfluidic devices such as those commonly produced by soft lithography methods (Figure 2), but with faster prototyping and simpler processing, easier fabrication of complex 3D geometries, the ability to fabricate circular channel cross-sections, and integrated fluidic interfaces. Furthermore, novel techniques were developed to fabricate double-sided or multilayer microfluidic devices that maintain the basic procedure of generating a Computer aided design (CAD) model, 3D-printed mold, and PDMS replica of the mold. These techniques, including alignment marks to precisely position molds (for example, in double-sided molding) or PDMS layers (for example, in multilayer assembly) as well as PDMS–PDMS bonding using a 3D-printed stamp, enable the design, fabrication, and assembly of complex microfluidic systems as shown in Figure 1f (Section 'Multisided and multilayer molding techniques').

### Single-sided molding techniques

Using 3D-printed molds, semicircular and fully circular channel geometries are easily fabricated (Figure 2); the high fidelity of PDMS transfer molding for features as small as 80 nm ensures that mold roughness is reliably transferred to the resulting PDMS^40^. Surface texture in ProJet™ 3000 multijet printing arises both from the interface between sequential rows of epoxy and from structural irregularities within a row. The interfacial texture resulted in a peak surface asperity of ~20 μm, measured by surface profilometry (Supplementary Figure S2c). Structural macroroughness, measured by Walczak *et al.*^24^, was 0.70 μm and 0.56 μm in the *x* and *y* directions, respectively, values comparable to those achieved in micromilling. Supplementary Figure S2b shows a PDMS component after release from the 3D-printed molds, with an enlarged view of the surface roughness. Although this value is comparable to microfluidics fabricated directly by 3D printing, transfer-molded PDMS components can produce narrower channels because interior cavities have the tendency to reflow during printing. In addition, surface roughness in 3D-printed devices is currently higher than in those fabricated by conventional soft lithography^23^. For further discussion, see Supplementary Material S1.2 (for surface roughness) and Supplementary Material S3.1 (for single-sided molding).

#### Glass ℓPDMS spin bonding

To create fully enclosed and tightly sealed microfluidic channels, a glass-bonding step is required^56^. However, because of the surface roughness of the 3D-printed molds, it was difficult to achieve a tight seal when bonding PDMS to glass using standard techniques such as oxygen plasma and ozone surface treatments. For this reason, specialized bonding techniques were necessary to finalize the microfluidic devices fabricated through the 3D PTM process. Although some surface roughness was reduced by performing a standard surface treatment (for example, in oxygen plasma) and then tightly clamping the two bonding surfaces together to mechanically compress the surface profile, this technique was unreliable and often led to broken glass during the curing stage (Supplementary Material S5.1).

A more reliable glass-bonding technique uses spin-coating uncured liquid PDMS (ℓPDMS) as both bonding agent and filler. ℓPDMS spin-bonding overcomes drawbacks associated with surface roughness from 3D printing and achieves a tight bond between the PDMS component and the glass slide (Figure 2d). First, ℓPDMS was spin-coated on a microscope coverslip (22×22×0.1 mm^3^, Fisher Scientific, Hampton, NH, USA) at 1800 Rotations/revolutions per minute (RPM) to achieve a thickness of 15 μm (Ref. 57). The ℓPDMS-coated slide was placed face up on a TexWipe TechniCloth® (Kernersville, NC, USA) resting on a hotplate at 95 °C for 70 s to partially cure the ℓPDMS, increasing its viscosity. The molded PDMS component was then placed bonding side down into the curing ℓPDMS, and *≈*20–30N downward force was applied to the component via a thick non-bonding glass side (Fisher Scientific 75×25×1 mm^3^) for 90 s. This thicker glass slide was used to apply pressure to equalize the distribution of the downward load and ensure that the PDMS bonded fully to the glass slide throughout its area. Next, the pressure was released, and the PDMS–glass bond was left to cure on the hotplate for an additional 5 min (Supplementary Material S5.2).

For double-sided microfluidic components, this process was repeated on the reverse side, with care taken to not fracture the fragile glass slide that had already been positioned during the first bonding step. Using a smaller glass coverslip for the upper surface, the upper glass slide could be bonded in a position that maintained access input and output holes. Although the ℓPDMS spin-bonding method worked for taller channels, for channels smaller than *≈*100 μm in height, excess ℓPDMS was sometimes forced into the channels, causing permanent blockages. This problem is mitigated using a 3D-printed stamp to selectively apply ℓPDMS only to non-channel areas of the PDMS device (Section 'ℓPDMS stamp bonding and multilayer rapid assembly'). Reliability of the bonding process can be further improved through the use of a dedicated bonding platform^19^ or through the use of a vacuum-bonding apparatus to provide consistent uniform pressure^58–61^. For more details on spin bonding and potential improvements, see Supplementary Material S5.3.

#### Integrated fluid inlets

Figure 2f shows a single-layer microfluidic device with integrated fluid inlets (diameter 0.55 mm), incorporated during mold design. These inlets simplify fabrication by eliminating the need for an additional hole-punching step. Six 20-gauge stainless steel interconnectors (Instech SC20/15, Plymouth Meeting, PA, USA, outer diameter 0.91 mm and length 15 mm) were easily inserted into the inlet ports for the connections to external fluidic pipes as shown in Figure 2f. Due to the tight seal of the steel couples against the 40% smaller inlet ports, the inlets were leak-resistant to pressures above 4 atm, the pressure at which the PDMS–glass bond delaminated when using untreated glass coverslips (Supplementary Material S5.2.2). 3D-printed guideposts at the corner of the mold assist with the removal of the PDMS without damaging narrow gauge inlets and outlets (Supplementary Material S2.2).

### Multisided and multilayer molding techniques

The increasing demand for microfluidics is particularly high for multilayered devices^62^. Multilayered fabrication allows for the implementation of more sophisticated and useful internal structures (including, valves or pumps) as well as reducing geometrical constraints by enabling fluidic detours and vias. Creating multilayer microfluidic devices using conventional techniques requires at least two lithography steps and one PDMS–PDMS bonding step (such as, for 'Quake' membrane valves)^63^, and can require up to four lithography steps and three PDMS–PDMS bonding steps (such as, for PDMS-based fluidic transistors)^64^.

**Figure 3:**
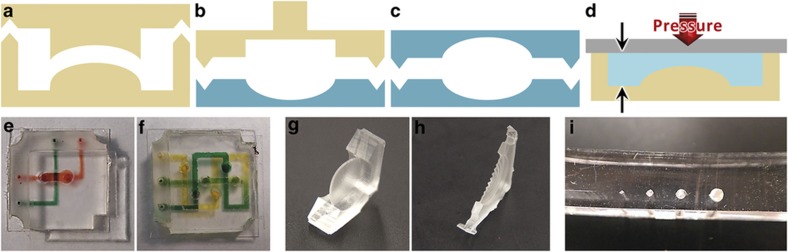
Double-sided molding techniques and results. Conceptual illustration of alignment marks: (**a**) mold–mold, (**b**) mold–PDMS, (**c**) PDMS–PDMS, and (**d**) PDMS height limiters. (**e** and **f**) Microfluidic devices with integrated fluidic vias: (**e**) simple overpass and (**f**) repeated crossover with mixing. (**g** and **h**) Membranes (350-μm thick) for fluid storage or hydrodynamic capacitance: (**g**) domed membrane (**h**) sinusoidal membrane for increased flexibility. (**i**) Fully circular channels fabricated by bonding two complementary components using integrated PDMS-PDMS alignment marks. PDMS, poly(dimethylsiloxane).

**Figure 4:**
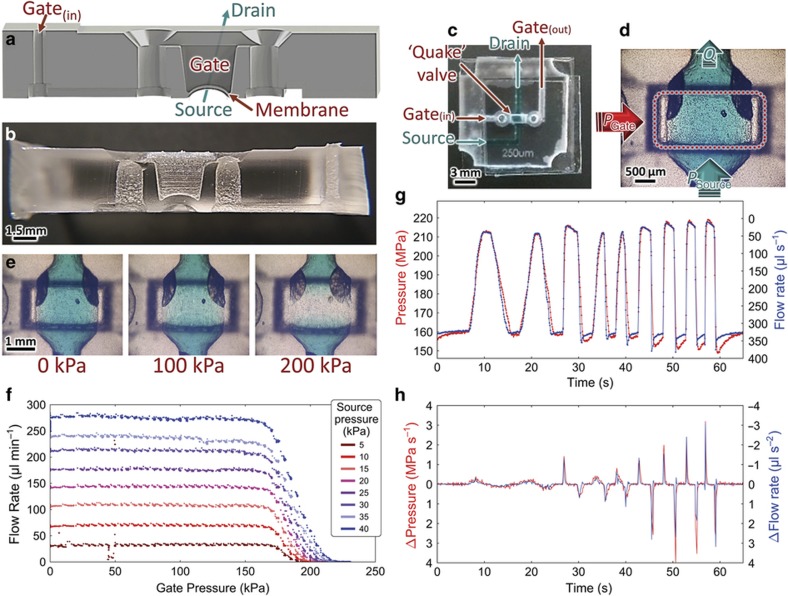
'Quake'-style membrane valves generated by single-step double-sided molding procedure. (**a**) Conceptual and (**b**) cross-sectional photograph of the membrane valve. (**c**) Top-down photograph, (**d**) microscope image illustrating the active valve region, and (**e**) microscope images of valve under various *P*_G_. (**f**) Valve characteristic curves under parametric *P*_S_ sweep. Further *Q*, *P*_G_ time series analysis: (**g**) flow rate compared with varying gate pressures and (**h**) rates of change of flow rate and gate pressures.

**Figure 5:**
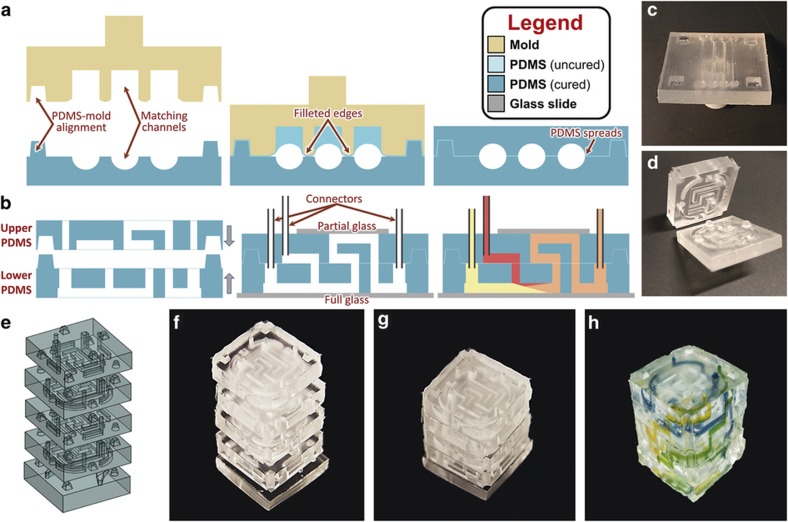
Rapid assembly of multilayer PDMS microfluidic devices achieved with 3D-printed molding. (**a**) Use of a 3D-printed stamp to selectively apply uncured PDMS to non-channel areas. (**b**) Conceptual illustration of alignment and assembly of multilayer microfluidic device with illustration of intralayer fluid flow. (**c**) Intruded stamp topography. (**d**) Second layer of six-layer device with corresponding stamp, which exhibits extruded stamp topography. (**e–h**) Rapid assembly of multilayer microfluidic device: (**e**) CAD model, (**f**) stacked individual layers, (**g**) assembled and bonded, and (**h**) fluid flow spiraling between layers and mixing. 3D, three-dimensional; PDMS, poly(dimethylsiloxane).

Multilayer construction is used largely to overcome the limits of traditional 2D microfluidic systems and to provide fluids with an extra degree of freedom. These 3D devices may be constructed either from PDMS components with features molded on more than one side (Figures 3 and 4), or with several layers of PDMS components (Figure 5). Although multilayer assembly is relatively common in traditional soft lithography (despite the aforementioned difficulties), double-sided molding is rare. Here, double-sided molding is accomplished through the use of in-mold alignment marks and can be used in preparation for multilayer assembly (for example, when constructing PDMS-alignment marks) or as a final device, in which case double-sided glass-bonding is performed to seal channels on both sides, leaving inlet/outlet holes uncovered on the upper surface (Supplementary Material S3.2).

#### Alignment marks

With 3D-printed molding, rapid assembly of multilayer microfluidic devices is easily achievable through the use of integrated alignment marks. Alignment marks can be used on the 3D-printed molds of the PDMS components, enabling multiple fabrication steps by allowing for the precise positioning of each layer without the need for a microscope. Figure 3a shows the four primary varieties of alignment marks: (i) mold–mold alignment marks, used for fabricating double-sided PDMS components; (ii) mold–PDMS-alignment marks, used in stamp bonding; (iii) PDMS–PDMS alignment marks, used for fabricating double-sided channels and assembling multilayer devices; and (iv) PDMS height limiters, used for controlling the ultimate thickness of a PDMS layer. For more detailed process flows and examples, see Supplementary Material S4.

#### Microfluidic vias

The ability to route fluid in three dimensions is frequently desired in microfluidics because it reduces geometrical constraints by enabling fluidic detours^65^. However, because these microfluidic vias are time-consuming and costly to fabricate (generally requiring a minimum of three lithography steps and one PDMS–PDMS alignment/bonding step), vias are not incorporated into microfluidic devices unless required for specific functionality.

With 3D-printed double-sided molding, however, fluidic vias are far simpler to fabricate^45,46^. Using double-sided molding techniques with columns that run from the bottom mold and fit into the upper mold (Figure 3b), smaller mold–mold alignment marks can be constructed that allow fluid to flow between the top and bottom face of a single layer of PDMS (Figure 1a) or between layers of bonded PDMS (Figure 5b), thus enabling multilayer microfluidic devices. For technical discussion, see Supplementary Material S3.2.1 for fabrication information and Supplementary Material S6.1 for a comparison of via manufacturing methods.

#### Thin membranes

Double-sided PDMS molding also enables the construction of integrated thin membranes. Nesting (but non-contact) mold features — created between the top and bottom molds — can be filled by ℓPDMS during vacuum-degassing, forming thin membranes upon curing. Figures 3g and h depict hyphenation problems membranes (domed and sinusoidal, respectively) that can potentially be used as fluidic reservoirs or capacitors. The sinusoidal corrugation lowers the effective spring constant of the membrane, allowing it to store more fluid. This technique has been used to generate membranes down to 200 μm thick, limited by surface interaction effects that interfered with PDMS curing^23^ and caused membrane rupture upon demolding. For further discussion on membrane uses, limitations, and design considerations, see Supplementary Material S3.2.2.

#### Double-sided channels

In certain microfluidic applications (such as, optofluidic lithography), it is useful to have components completely surrounded by PDMS^66^ or channels that have 360° curvature^67,68^. Figure 3i shows an image of a fully rounded microfluidic channel fabricated using the 3D PTM process that avoids some of the difficulties of current techniques for generating fully rounded channels. For further fabrication results and motivations, see Supplementary Material S3.4.

#### Membrane valves

Another common requirement in multilayer microfluidics is membrane valves, which use pneumatic or hydraulic pressure in one fluid layer to moderate fluid flow in a secondary layer^65^. Commonly, these membrane (or 'Quake') valves are two-layer constructions that use multiple pneumatic inputs to control complex arrays of microfluidic reactors, although some studies use multiple layers to implement valves with active control or integrated pressure gain^69,70^. Fully 3D-printed microfluidic systems with valving mechanisms have also been developed^22,71–73^.

In this work, we fabricated a membrane valve using a single-step double-sided microfluidic molding technique by linking a detour via with a thin membrane in an upper layer (Figures 4a and b). The membrane was 350 μm thick, and the lower channel was 500 μm deep (for schematics, see Supplementary Figure S13). Although standard membrane valves require a photoresist reflow step during manufacture to allow the bottom layer to close fully, we were able to implement a rounded lower channel directly from the CAD file. To characterize the closing behavior of membrane valve, we ran a series of pressure sweeps (Fluigent MFCS–EZ, Villejuif, France) and measured (Fluigent FlowUnit L) the resulting source-drain flow rate (*Q*): gate pressure (*P*_G_) was increased smoothly and source pressure (*P*_S_) was increased parametrically. The valve began closing at 160 kPa, was fully closed by 220 kPa, and exhibited a nearly linear response during the transition (Figure 4f). Further, *P*_S_ did not substantially affect the *P*_G_ of the initial drop in *Q*. Finally, to demonstrate the response time of the membrane valve within the closing pressure window, we manually cycled the gate pressure at various speeds and compared the pressure and flow rate response curves (Figure 4g). Note that the vertical axes have been rescaled and shifted to illustrate the high degree of qualitative agreement between pressure and flow rate. The time-differential response curves (Figure 4h) illustrate the rapid response time of flow rate to changes in gate pressure.

### ℓPDMS stamp bonding and multilayer rapid assembly

Double-sided PDMS molding can be completed by spin-bonding to glass; however, fully multilayer microfluidic devices (created by 3D-printed molding or conventional soft lithography) require a PDMS–PDMS bonding step. For 3D molded PDMS components, we have developed 3D-printed stamps to selectively apply PDMS as a more consistent bonding technique, as shown in Figure 5a. The stamps can easily be designed and printed from the original CAD drawings to selectively apply uncured ℓPDMS only to non-channel areas of the PDMS component. These stamps can contain features that intrude (Figure 5c) or extrude (Figure 5d) from the plane of the stamp, which allows the stamp to be used on numerous PDMS topographies. Using ℓPDMS stamp bonding, multilayered microfluidic devices are easily assembled, allowing fluid flow within or between the various layers (Figure 5b). In addition, previously discussed techniques (including, alignment marks, integrated inlets/outlets, and variable channels) can be used in conjunction with rapid assembly. Including the 40 min baking time, ℓPDMS stamp bonding allows a six-layer microfluidic device (Figure 5e) to be assembled and bonded in under an hour.

To bond two PDMS components with ℓPDMS stamp bonding, ℓPDMS was first applied to a TechniCloth wipe, and the stamp was dipped in the PDMS. For extruded stamps, a copy of the PDMS component was used as an applicator (that is, a template stamp used to transfer ℓPDMS to the extruded stamp topographies). Next, the stamp was 'blotted' with a clean TechniCloth to remove excess PDMS. The stamp was then pressed lightly (≈5−10 N) into its complementary PDMS component to deposit a thin, uniform layer of ℓPDMS on the 4 cm^2^ PDMS component. Finally, the two PDMS components were pressed together and clamped in place to prevent shifting during curing. Clamping was performed with a 1 ½" C-clamp and was judged to be sufficiently secure when the visual roughness disappeared (as ℓPDMS filled the empty spaces resulting from the roughness). Finally, the devices were cured at 80 °C for 40 min, resulting in a complete multilevel PDMS device. For a more extensive discussion of stamp-bonding techniques, see Supplementary Material S5.3. Note that these precision stamp-bonding techniques may prove useful for bonding disparate materials that would otherwise require harsh plasma treatments^74–76^ or for the precision placement of cells or other biomaterials^7^.

## Discussion

In this work, we presented a novel method for rapidly manufacturing elastomeric microfluidic devices using 3D printed transfer molding (PTM). Although this process was limited by the resolution and surface roughness of the ProJet™ 3000 multijet printer, the technique was able to reliably produce enclosed channels as narrow as 100 μm. In conjunction with a spin-coated ℓPDMS glass-bonding technique, designed to counteract the effects of the mold’s surface roughness, this method can produce single-layer microfluidics more flexibly than those produced in standard soft lithography fabrication processes. In addition, the transfer-molded microfluidic devices are enhanced by numerous design elements, not limited to the following: controllably non-rectilinear channels, integrated inlets and outlets, vias and thin membranes, and integrated alignment marks, techniques that can be applied more generally across the 3D printer across 3D printer models and methodologies.

Furthermore, PTM techniques are far more versatile than merely replicating existing soft lithography; by incorporating newly developed alignment marks and ℓPDMS stamp-bonding, this process can produce complex multisided and multilayer microfluidic devices with ease. Single-step double-sided manufacturing (in which features are patterned on both sides of the PDMS components) enables features such as microfluidic vias and membrane valves. Microfluidic vias, which allow fluids to flow in three dimensions, reduce geometrical constraints and were fabricated here with diameters as narrow as 550 μm to hold 20-gauge catheter couples. Membranes (350 μm) were used to produce microfluidic valves with an actuation pressure of 200 kPa. Furthermore, by combining the double-sided manufacture method with novel custom 3D-printed stamps, rapid assembly of multilayer microfluidic devices was demonstrated. The 3D-printed stamps can selectively apply a thin layer of ℓPDMS adhesive—used to compensate for surface roughness—to non-channel areas, preventing the PDMS from clogging the final microfluidic device. Furthermore, because adhesive bonding techniques have been used to bond disparate materials, we expect the stamp-printing techniques introduced here to remain relevant past the point at which printers have sufficient resolution to mitigate roughness issues. In summary, the 3D PTM process allows the rapid fabrication of multilayered microfluidic devices, combining the flexibility and speed of emerging 3D printing technology with the well-known mechanical and biological properties of PDMS favored by microfluidic researchers.
