# Supplementary material for: Rapid assembly of multilayer microfluidic structures via 3D-printed transfer molding and bonding
Source: Microsyst Nanoeng. 2016 Nov 21;2:16063. doi: 10.1038/micronano.2016.63 (PMC6444730; doi:10.1038/micronano.2016.63)
Supplement: Supplementary information [file micronano201663-s1.pdf]

Supplementary file

Rapid assembly of multilayer microfluidic structures via 3D-printed transfer molding and bonding

Casey C. Glick[1,2], Mitchell T. Srimongkol[2*], Aaron J. Schwartz[2*], William S. Zhuang[2*], Joseph C. Lin[2*], Roseanne H. Warren[2,3], Dennis R. Tekell[2], Panitan A. Satamalee[2] and Liwei Lin[2]

Microsystems & Nanoengineering (2016) **2,** 16063; doi:10.1038/micronano.2016.63; Published online: 21 November 2016

S1 PROJET™ 3000

S1.1 Printing and material details

The ProJet™ 3000 printer (3D Systems, Rock Hill, SC, USA), printing in UHD mode, first lays down a 2 mm thick layer of VisiJet® S100 Support Material (sacrificial wax), resulting in smooth bottom surfaces of devices. Next it lays down alternating amounts VisiJet EX200 epoxy and S100 support in 35 μm layers. The print is deposited on an aluminum tray and can print within a volume of $127 \times 178 \times 203$ mm^3 with a resolution of ≈0.05 mm for extruded parts. 3D Systems cites an elastic modulus of 1.159 GPa, although comparison of experimental and simulated tests performed by Sochol *et al.* suggest the proper modulus, post wax removal, is closer to 1/10 or 1/20 that value[1]. EX200 devices begin to soften past their glass transition temperature of 52.5 °C and become milky, although this effect reverses upon cooling of the 3D printed part. Softness continues to increases as temperatures are raised, until by 150 °C the pieces can be easily and permanently deformed with even gentle pressure from metal tweezers. Although care must therefore be taken not to place too much concentrated force on the device in a hot bath, we find below 80 °C, the devices were not deformed by the tweezers when moving them in and out of their cleaning baths. More caution must be taken to preserve fine cantilevered features during cleaning, as these features can break at any temperature. Because many devices had at least one high-aspect feature (e.g., input/output) break by 10 moldings due to handling error during demolding, we redesigned the input/output ports to be 50% thicker at the base and to fillet high aspect features at the base to reduce stress points; these redesigns limited feature degradation. We did not notice significant degradation of features with aspect ratios of less than 10:1.

The HD Systems proprietary epoxy blend, VisiJet EX200, consists of a base of 20-40% proprietary urethane acrylate oligomers' and 15-35% ethoxylated bisphenol A diacrylate, as well as 1.5–3% tri(propyleneglycol) diacrylate as a curing agent[2,3]. The sacrificial wax support material consists of a hydroxylated wax[4] with a melting temperature of ≈55 °C Based on experiments, S100 is fully soluble in Bayes mineral oil and is mostly soluble in most brands of vegetable oil. For more information, see the documentation on the 3D Systems website. Links for the ProJet™ HD3000 3D Printer documentation are listed in the citations for this work, including the ProJet HD 3000 brochure, the EX200 Resin table of values, the EX200 MSDS, and the S100 MSDS[2–5].

S1.2 Surface roughness and roughness mitigation

A consistent problem with the ProJet™ 3000 printer is the degree of surface roughness it creates; peak asperities of 20 μm occur every 0.5 mm, and the general RMS surface roughness was 0.70 μm and 0.56 μm in the X and Y directions, respectively[6]. This roughness is largely a feature of the printer's integrated resolution and will increasingly become a non-issue as printers improve. However, the roughness is transferred to the PDMS (Sylgard 184 Elastomer Kit, Dow Corning, Midlind, MI, USA) surface after molding. Attempting to mitigate roughness, we engaged in numerous experiments to attempt to reduce the overall surface roughness of the mold to allow for smoother Glass-PDMS and PDMS-PDMS bonding in later steps. Ultimately, all attempted techniques resulted in either no effect or a worsening of roughness, and we were forced to use liquid PDMS (ℓPDMS) as a binding agent to overcome the surface asperity. Note that when the molded PDMS is placed in liquid PDMS, degassed, and baked, the original vanishes, indicating that the optical opacity on the PDMS component is purely a surface artifact resulting from the macroroughness of the mold and not from chemical transfers from the molding material. Accordingly, the PDMS macroroughness disappears during ℓPDMS spin and stamp bonding, as the ℓPDMS fills vacancies in the surface texture.

The effect of surface roughness on optical properties has been well studied[7–10] and may be partially summarized by the Rayleigh criterion, which is approximately

$$Ra_t = \frac{2\pi}{\lambda}\ \sigma_h\ \frac{|n_1 \quad n_2|}{2}$$

for normal transmission from one dielectric to another, where λ is the wavelength, σ_h is the surface RMS (root mean square) height, and n_1 and n_2 are the dielectric constants of the two media, and where an optically rough material is characterized by $Ra_t > \frac{\pi}{2}$ (Ref. 10). Choosing $n_1 = 1$ for vacuum, $n_2 = 1.4$ for PDMS[11], and $\lambda \approx 500$ nm for the average wavelength of visible light, PDMS will appear optically rough for $\sigma_h > 625$ nm. Notably, the Rayleigh criterion is similar to the ≈600 nm RMS roughness of the 3D printed molds observed by Walczak *et al.*, explaining the observed optical opacity[6].

S1.2.1 Chemical Roughness Mitigation

(I) Solvents (IPA, Ethanol, Acetone, Goo Gone® (Goo Gone® Gurnee, IL, USA)) Solvent use resulted in swelling (to various degrees) of the mold surface and a buildup of a white powdery layer. The minimal

[1]Department of Physics, University of California, Berkeley, CA 94720, USA; [2]Department of Mechanical Engineering, University of California, Berkeley, CA 94720, USA and [3]Department of Mechanical Engineering, University of Utah, Salt Lake City, UT 84112, USA.
Correspondence: Casey C. Glick (glick.casey@gmail.com) or Liwei Lin (lwlin@me.berkeley.edu)
*These authors contributed equally to this work.

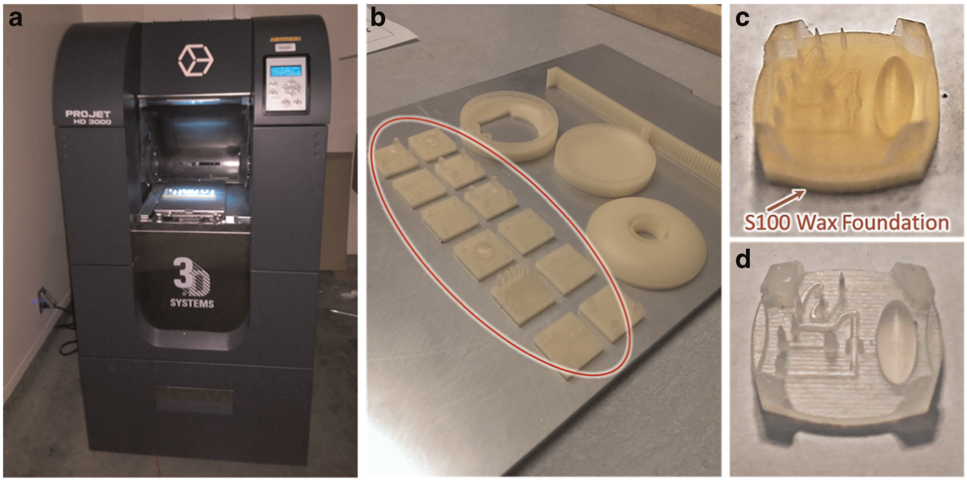

Figure S1 ProJet3000 3D Printer basic output. **(a)** The printer itself with a completed print sitting on the print tray. **(b)** Aluminum print tray with 3D-printed molds still attached by wax layer (circled). For cost and time-saving considerations, this print run was shared with other users of the CiBER laboratories. **(c)** A single 3D printed mold before wax has been removed and **(d)** mold after the removal of wax.

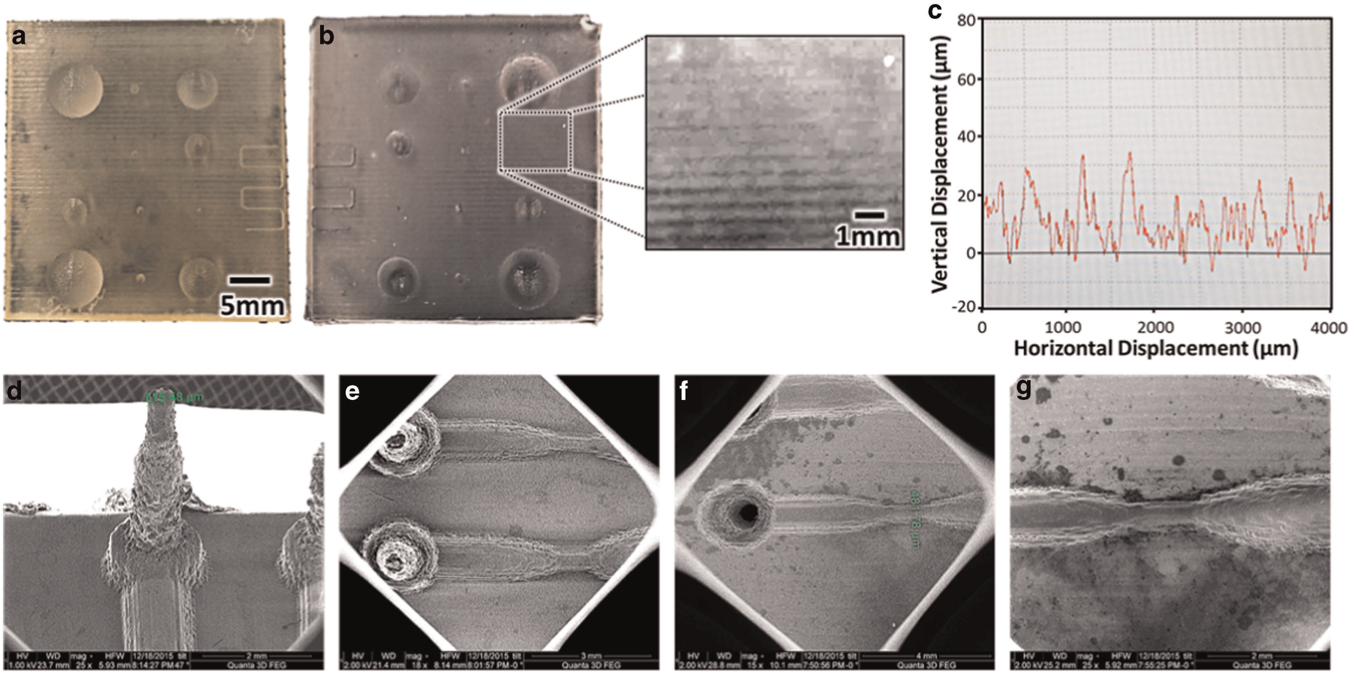

Figure S2 Surface roughness of 3D printed mold and transferred PDMS. Top: Sample device to test surface roughness. Bottom: SEMs of Single layer device (Supplementary Materials S3.1) (**a**) 3D printed mold with visible roughness and (**b**) corresponding molded PDMS with magnified insert highlighting roughness. (**c**) Profilometer measurements of 3D printed mold showing ≈20-30 μm surface asperity every 0.5 mm. (**d**) Angled view of single layered mold showing roughness of integrated inlet generation pillar (diameter 515 μm. (**e**) Top view showing layering pattern of channel mold. (**f**) Corresponding molded PDMS showing high fidelity of transfer of surface roughness, and (**g**) zoomed view. Note that the dark splotches in (f) and (g) are artifacts from improper conductive surface treatment of the PDMS before the SEM scans.

wax removal was not enough to justify the damage to VisiJet material.

(II) H_2O_2 (1M) and NaOH (1M) Neither H_2O_2 (Sigma Aldrich, St. Louis, MO, USA), or NaOH (Sigma Aldrich) had any noticeable effect on mold or on wax.

(III) H_2SO_4 Sulfuric acid (H_2SO_4, Sigma Aldrich) had no noticeable effect on mold in concentrations lower than 50% H_2SO_4. At very high concentrations ($>98\%$), H_2SO_4 began to discolor the surface of the molds, taking on a thick slimy texture. This layer could be rinsed off, leaving the mold with slightly reduced surface texture. Ultimately, H_2SO_4 led to slight reduction in aspect ratio and corner definition and did remove the large print lines from the 3D printer, but caused an overall increase in surface roughness, possibly due to the remove of long polymer units from the mold. The new surface roughness was more uniform, but the effect was not significant enough to make up for other difficulties with the method. We postulate that the effects are only present at very high acid concentrations because the molds are only affected by

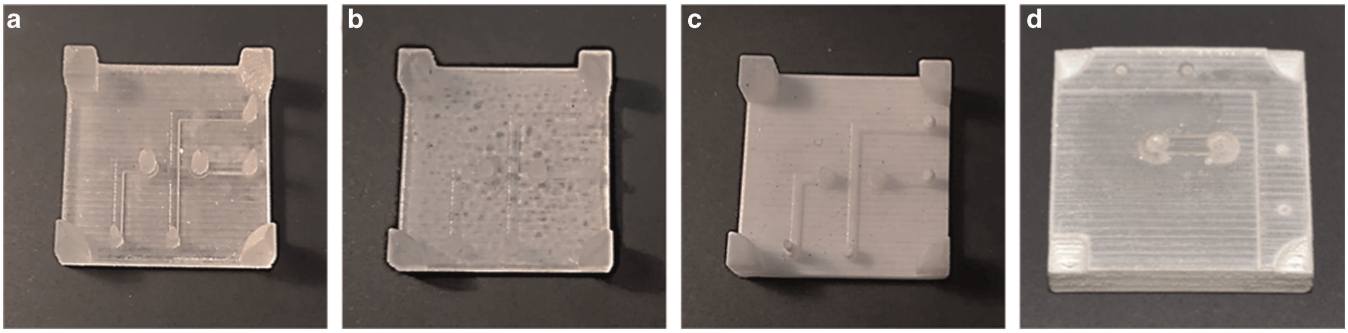

Figure S3 Different manifestations of color and texture change seen on 3D printed transfer molds: **(a)** Standard color of VisiJet200 material after cleaning, **(b)** mottled visual texture, **(c)** all-white surface coloration, and **(d)** powdery texture (and surface curvature) after acetone absorption.

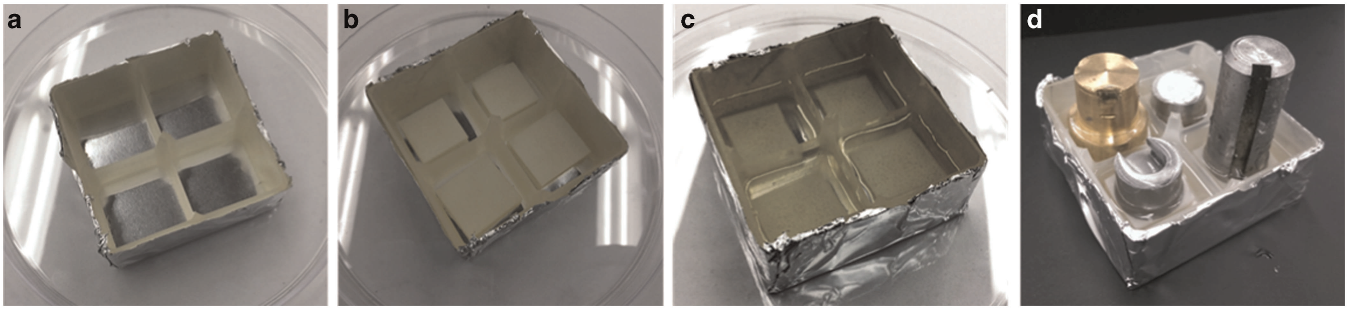

Figure S4 3D-printed molding containers and molding techniques. An empty molding container is (**a**) wrapped with aluminum foil to prevent PDMS leakage. (**b**) Molds are placed into the container and (**c**) PDMS is poured. (**d**) For double-sided molds, metal weights between 50–100 g are placed on the molds and the whole device is degassed.

the well-known dehydrating properties of the H_2SO_4 (most evident at very low pH), and not by the corrosive effects due to protonation.

(IV) HNO_3 (0.1M) Nitric Acid (HNO_3, Sigma Aldrich) did not cause surface effects, but instead was absorbed fully into the 3D printed mold, causing a systemic and permanent mold color change to orange. Additionally, the mold became highly brittle and could be easily broken.

S1.3 Color and visual texture change

The visual appearance of the 3D printed molds made of VisiJet200 material does not remain constant over the device lifetime. When the devices first emerge from the printer, they are a deep golden yellow color, with higher degrees of orange for devices exposed to more UV light (Figure S3a). During the ProJet manufacturing process, each applied layer (≈35 μm) is followed by an exposure of UV light to cure the layer. Thus, print runs that have tall devices will experience far more exposure to UV than print runs that contain short devices. Additionally, molds left out where they can be exposed to sunlight will eventually deepen in color, provided they are exposed to no other processes.

Once the molds are baked with curing PDMS, they begin to take on a spider-web appearance (Figure S3b). While the physical texture is not noticeably changed, the visual texture alters noticeably, sometimes making it difficult to identify the specific mold, as the spider web texture camouflages physical features. We are unsure of the specific reason for this color change, as the time scale for the change may be radically different for different devices. However, devices that have been PFOTS treated may undergo the changes more rapidly.

Frequently, but unpredictably, the devices may take on a solid off-white color and remain that through repeated moldings. We are unsure what causes this transformation, but generally welcome it because the devices are far easier to identify once the surface takes on a solid visual texture (Figure S3c). If the molds are placed in a solvent such as acetone, IPA, or commercially available solvents like GooGone®, the surface of the device will pick up a powdery white texture which is not generally removable, even if the mold is thoroughly washed in oil or soapy water and/or baked in the oven to facilitate the evaporation of solvents (Figure S3d). Additionally, the molds pick up a curvature from the swelling resulting from the solvent absorption. This particular color change can therefore be avoided.

S2 MOLDING TECHNIQUES

S2.1 Molding containers

PDMS soft lithography processes tend to waste PDMS, which is often reduced by placing the mold in a foil-wrapped container. Here, we present a set of 3D-printed molding containers (fabricated, cleaned, and PFOTS treated like standard molds) which hold the 3D molds and PDMS and reduce the PDMS waste volume. First, the mold containers are wrapped with aluminum foil (Figure S4a), after which the molds are placed within the container (Figure S4b). Next, 20-30 g degassed PDMS is poured on the molds (Figure S4c) and the molds are degassed a second time. For double-sided molds, pieces of scrap metal weighing between 50 and 100 g are placed on top of the upper mold (Figure S4d) to prevent bubbles from dislodging it during the degassing phase. Finally, the entire apparatus–molding container, molds, and uncured PDMS–is placed on a dish and baked. For double-sided

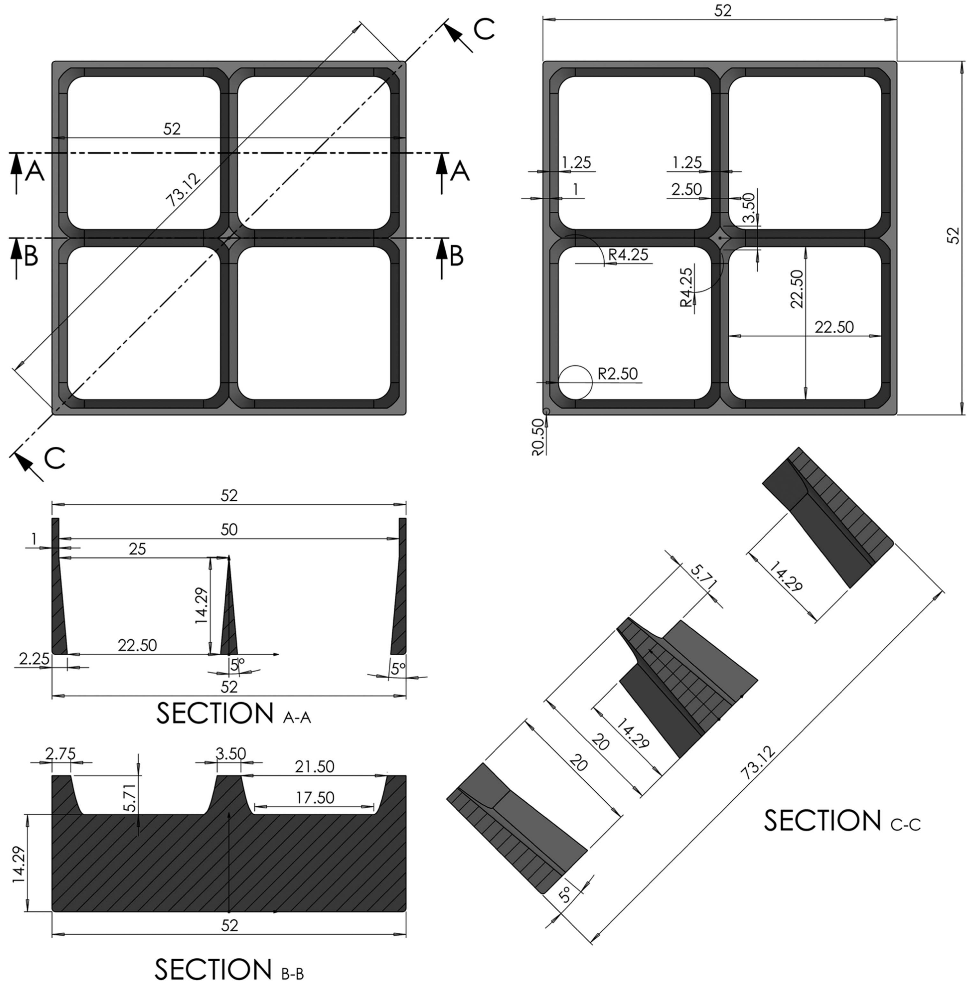

Figure S5 Schematic diagrams of 3D printed molding containers with horizontal and diagonal cross-sections.

molds, the loading/degassing process may be combined with a vacuum-assisted transfer procedure[12], which could further reduce PDMS waste.

Figure S5 summarizes the key geometries of the 3D printed molding container. Each molding container has room for four mold dies, occupying a floor space of $22.5 \times 22.5\ mm^2$, fitting the standard 3D printed mold size of $20x20\ mm^2$; larger molds may be accommodated by larger mold containers. The design shown in Figure S5 requires only an additional 1.2 g PDMS per cm pour depth (0.7 cm for single-sided molds, 1 cm for double-sided molds), plus PDMS poured beyond the mold height. Each mold die is drafted outward at 5° to allow for the easy ejection of the PDMS and mold after curing. Waste may be further reduced by (i) decreasing draft angle, (ii) decreasing floor space, and (iii) combining multiple devices into larger molds.

S2.2 Demolding techniques

After the devices are fully cured, the PDMS devices must be demolded, which proceeds in three main steps: (1) releasing the PDMS and mold from the molding container, (2) cutting away excess PDMS, and (3) removing the PDMS device from the mold itself.

(I) Releasing device from molding container After the molding container and PDMS cool, the metal weights are removed (this may be easier to do at an angle) so the device can be easily inverted. This will leave a visible vacancy at the top of the mold (Figure S6b) which will be eliminated when the component is fully demolded. Next, a series of shallow cuts are made on the bottom of the molding container, tracing the outline of each square (Figure S6a). These scorings separate the PDMS which is surrounding the mold from PDMS which has cured underneath the molding container. The aluminum foil may be removed during this step, but the removal is not strictly necessary and may be removed later (Figure S6f). Next, the mold container is turned upside down and the PDMS die is ejected (Figure S6c). Note that using the metal weights to push helps to eject the die.

(II) Removing excess PDMS At this point, the independent molds are surrounded in excess PDMS, which forms a truncated pyramid (the angle of the PDMS makes it easier to eject these dies). If the device is molded on both sides, cutting free the excess PDMS is relatively simple; the side of the molds is used as a guide for the scalpel which traces the sides. For single-sided devices, the device is placed PDMS-side down onto a cutting board and pressed downward to place the top surface of the die in full contact with the cutting board (there will be some meniscus on the PDMS from contact with the side of the molding chamber). Then, again using the edge of the mold as a guide, the scalpel cuts away the excess PDMS (Figure S6g). Lastly, for either device design, excess PDMS may be peeled from the far side of the mold surface, as it is no longer in contact with the main body of the mold.

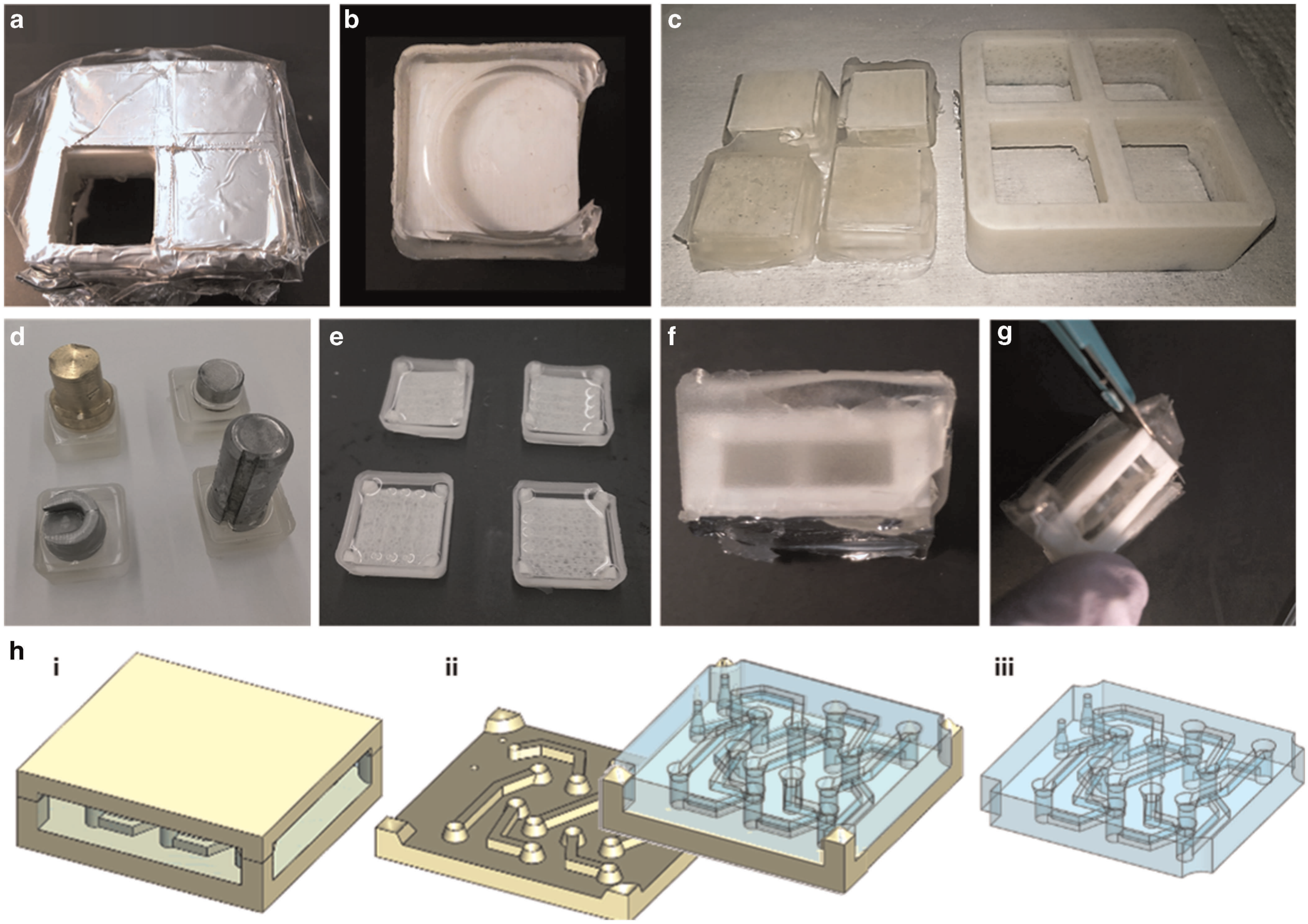

Figure S6 Removing PDMS devices from molding containers and molds. **(a)** Molding containers are scored at the bottom to release individual dies which are **(b)** pushed out to remove the mold and excess PDMS. **(c)** Molding container and individual dies side-by-side **(d)** Dies with metal weights still attached, and **(e)** Single-sided mold dies. To cut the mold loose, **(f)** an individual die is placed on its side, and **(g)** PDMS is cut loose with a scalpel, using the mold as a cutting guide. *Bottom* **(h)** Schematic diagram for removal of PDMS from double-sided mold: (i) released mold with cured PDMS, (ii) top mold removed, and (iii) PDMS component separated from mold.

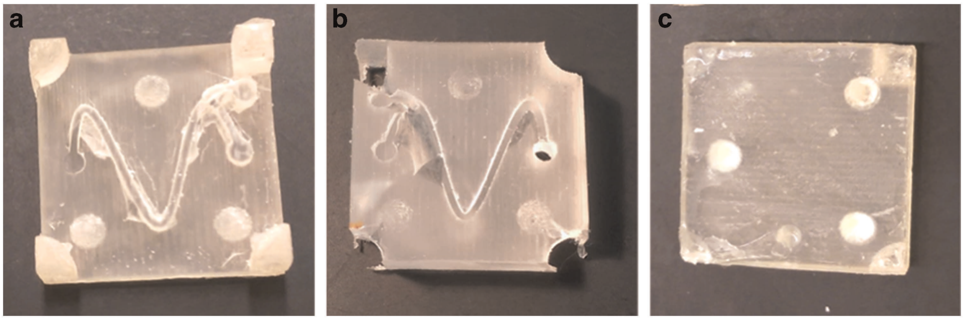

Figure S7 Problems resulting from incorrect application of PFOTS silanization. (a-c) Insufficient silanization: **(a)** Bottom mold, **(b)** Resulting PDMS, and **(c)** upper mold. Note how PDMS has remained attached to the lower mold by tearing from the bulk device.

(III) Removing PDMS from mold Once the excess PDMS has been cut free, the main PDMS device may be removed from the mold (Figure S6h). Depending on the mold design, this may require more or less care. If the mold is relatively simple and compact, the PDMS may be peeled off directly without worrying about mold damage. If narrow vias or alignment marks have been used, the PDMS must be removed perpendicularly from the surface. If numerous such features are used, the PDMS must be loosened from the surface a bit at a time and then slid from the mold by rocking the device back and forth as it is moved upward.

If a double sided mold is used, first, the top mold must be removed. In this project, we designed the top molds to have relatively minimal extruded features, meaning that the top mold

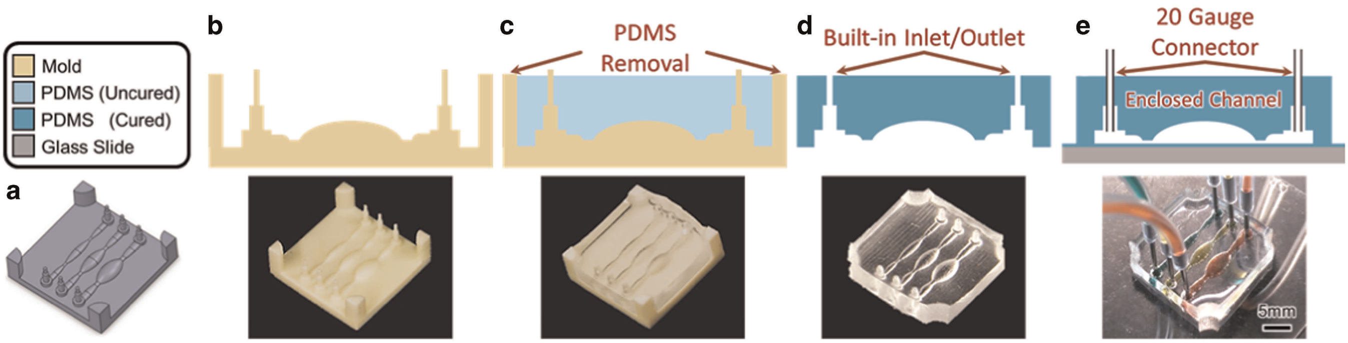

Figure S8 Illustration of the 3D printed transfer molding technique and process for single-sided device. *Top:* conceptual images. *Bottom:* Photographic images. **(a)** Mold is designed in CAD program such as SolidWorks™ **(b)** Mold is 3D Printed from CAD model, treated, and covered with PDMS which is then cured. **(c)** Excess PDMS is cut away using a scalpel and the edges of the mold as a guide. **(d)** Cured PDMS is removed from the mold using the guide-posts to assist manual removal. The PDMS device has integrated inlets and outlets, and is **(e)** bonded to glass to create enclosed, arbitrary channels. Stainless steel fluidic connectors can be directly inserted to the system via the integrated fluid inlets The PDMS structure can be bonded onto a glass substrate to make the enclosed channels and reservoirs system.

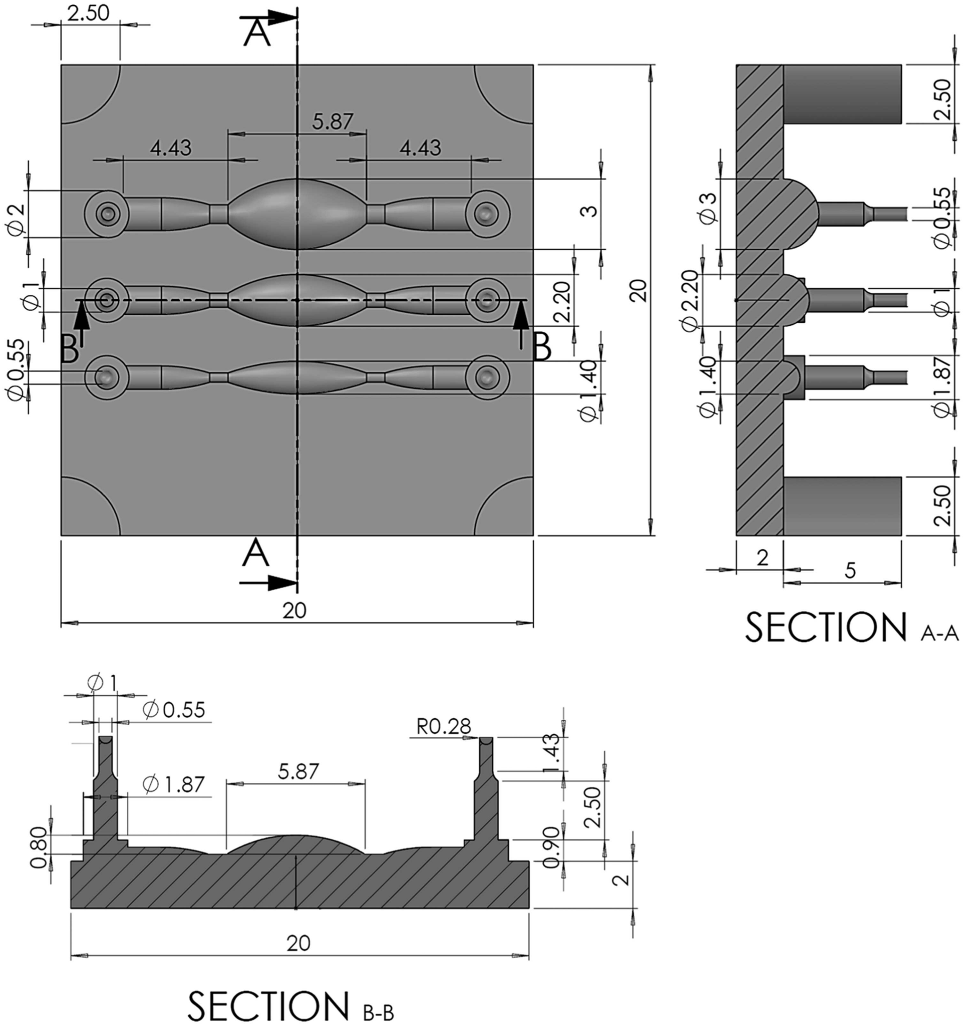

Figure S9 Schematic diagrams of single-sided mold with two distinct cross-sections illustrating the channel profiles.

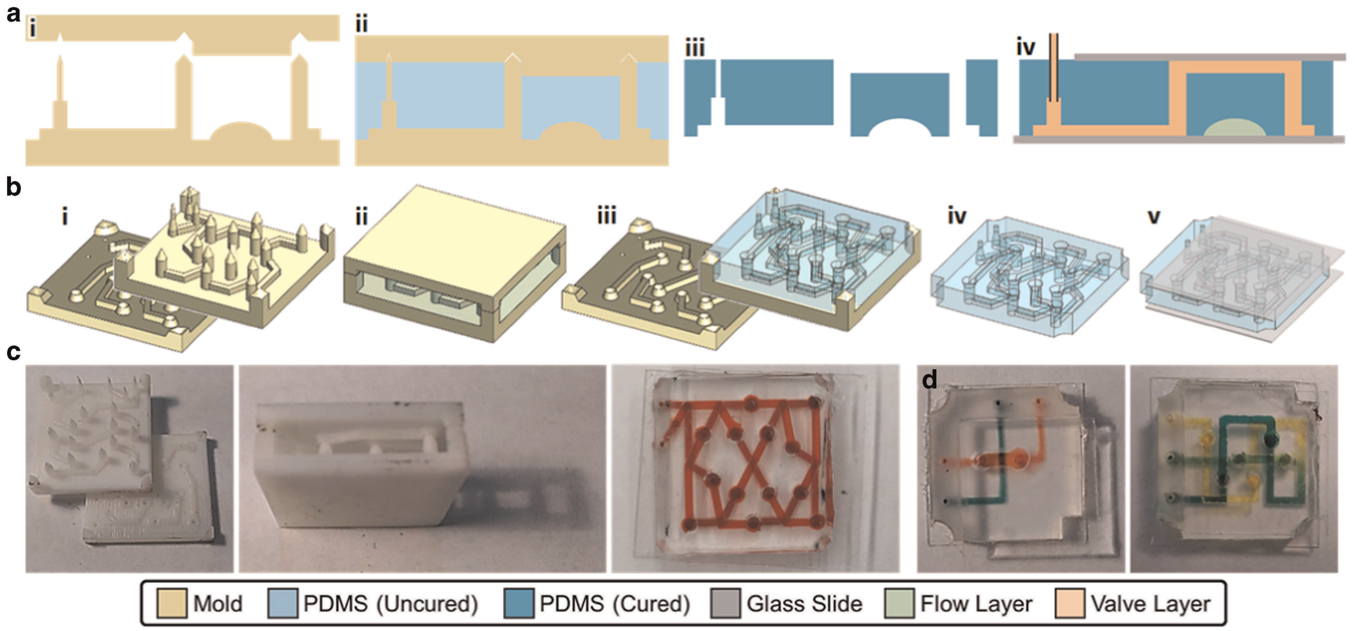

Figure S10 Fabrication flow for arbitrary vias constructed through double-sided transfer molding with integrated fluidic inlet. **(a)** Conceptual fabrication flow for Celtic Knot-inspired via device: (i) upper and lower molds, (ii) molds filled with PDMS, (iii) demolding by removing upper layer first, (iv) PDMS device, and (v) glass bonded device. **(b)** Fabrication photographs for Celtic Knot-inspired via device (i) upper and lower molds, (ii) assembled molds, and (iii) Knot device with fluid flow. **(c)** Simple overpass via-based PDMS device and **(d)** vias enabling repeated crossover and mixing.

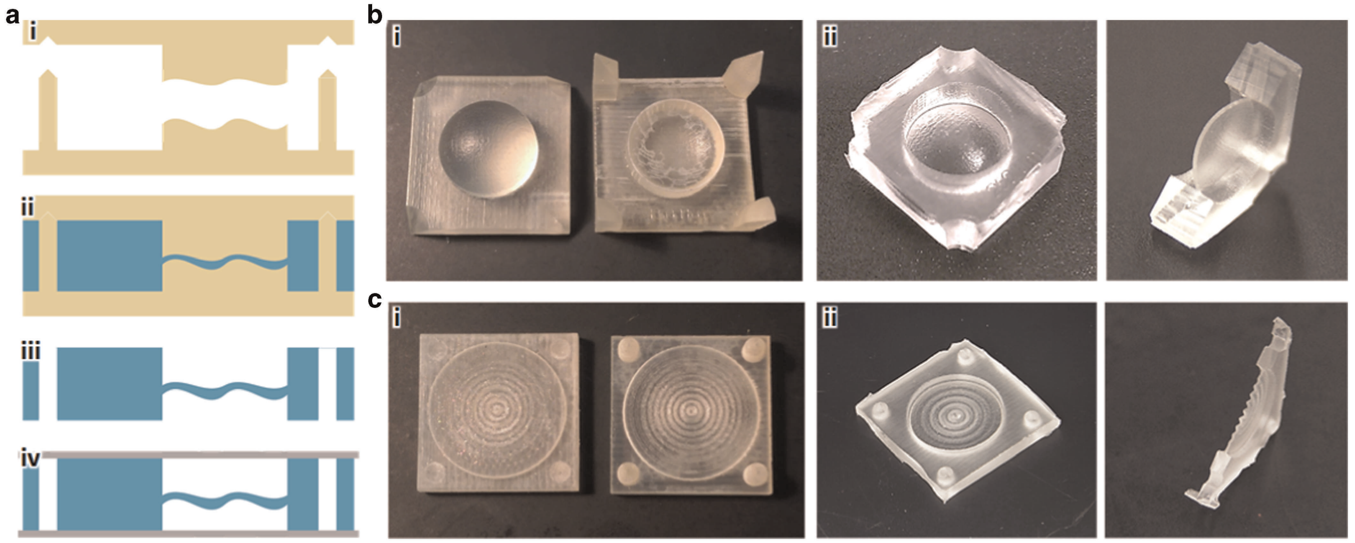

Figure S11 Techniques for fabricating thin membranes using 3D printed transfer molding. **(a)** Process flow for generating arbitrary thin membrane, including alignment marks which insure proper membrane nesting. **(b)** 300 μm domed membrane: (i) Molds, (ii) Isometric view, and (iii) side view **(c)** 300 μm sinusoidal membrane: (i) Molds, (ii) Isometric view, and (iii) side view. The sinusoidal pattern allows for more deformation without increasing the thickness or surface area[13].

could be leveraged off by getting a fingernail under one corner and peeling off at an angle. Note that if the mold's silanization treatment has begun to wear off, this may be difficult.

After the PDMS has been demolded, it must be inspected for connected strands of PDMS that were not effectively removed during the removal of excess PDMS. These may interfere with bonding if present, and so must be cut loose. Bonding problems may be alleviated by placing a small extruded lip on the surface of the mold (resulting in an intruded lip around the edge of the PDMS) which makes it easier to remove these edge beads of PDMS. For more details on bonding problems, see Supplementary Materials S5.2.1.

S2.3 Mold silanization

Performing proper PFOTS silanization treatments is important for the proper functionality of the microfluidic molding process; however, the process is relatively smooth within reasonable tolerances. Insufficient silanization (less than 10-15 min) causes the most obvious problems (Figure S7), resulting in the mold

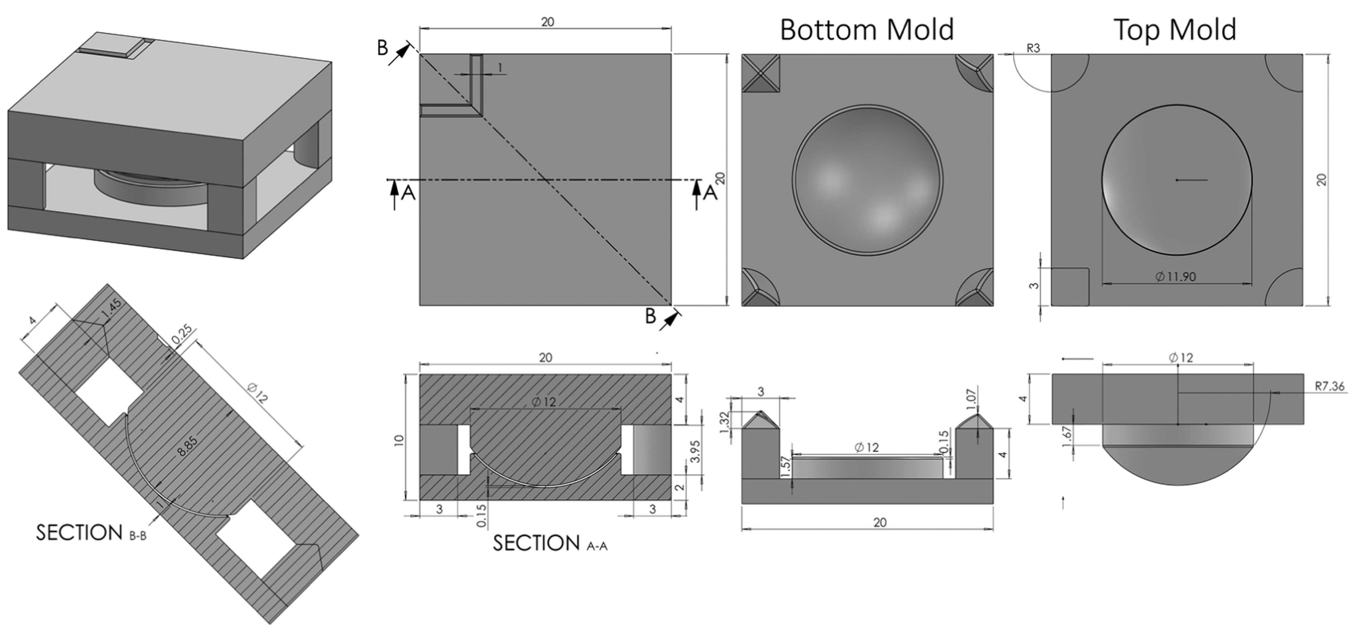

Figure S12 Schematic diagrams of 350 μm domed membrane molds, both nested and individually.

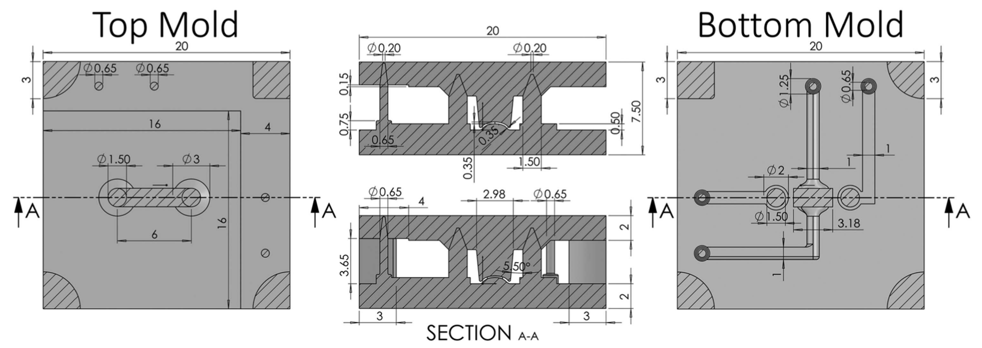

Figure S13 "Quake" valve mold schematics, showing top and bottom molds, as well as nested molds. Both cross-sectional views are of the identical view and location, excepting that the bottom cross-section shows the full depth-of-field and therefore illustrates the alignment marks in the background.

bonding permanently to parts of the PDMS. When this happens, PDMS will tear from the mold rather than peel away nicely. Additionally, because the PDMS has bonded permanently to the mold (and will have permeated the near surface of the mold) this process is not effectively reversible, even when silanization is reapplied.

Excessive silanization (over 1 hour exposure or larger amounts of PFOTS) may also cause problems as it prevents the curing of PDMS within 1 mm of the mold surface for all but extreme bake times (e.g., over one day). After demolding, the PDMS will eventually cure, but because the surface was still relatively liquid upon demolding, many fine features may be lost, including certain channel designs and membranes. Because the effect is similar to when liquid is absorbed into the 3D printed material, we believe that the PFOTS interferes with the cross-linking of PDMS.

S3 SINGLE AND DOUBLE SIDED FABRICATION

This section explores the techniques discussed in Supplementary Materials **S2.1** and Supplementary Materials **S2.2** in further depth, covering more explicit process flows, technical analysis, and dimensions for each device category.

S3.1 Single sided molding

The single-sided microfluidic device discussed in section 'Single-sided molding techniques' and depicted in Figure 2 was fabricated from a CAD model (Figure S8a) via the 3D printing process (Figure S8b) and PDMS was applied, cured (Figure S8c), and released from the mold (Figure S8d) by means of the PDMS molding steps described in Methods and Materials. Integrated fluid inlets (diameter 0.55 mm) can be easily incorporated to the device through the mold design, further simplifying fabrication by eliminating the need for an additional hole-punching step. Six 20-gauge stainless steel inter-connectors (Instech SC20/15, outer dimeter 0.91 mm and length 15 mm) were easily inserted into the inlet ports for the connections to external fluidic pipes as shown in Figure S8e. Due to the tight seal of the steel couples against the smaller integrated inlets were leak-resistant to pressures of up to 4 ATM. The PDMS device was then bonded to a glass slide to created closed microfluidic channels.

The microfluidic device consists of three microchannels with elliptical-shape reservoirs of dimensions 1.5x5.9 mm^2, 1.1x5.9 mm^2 and 0.7x5.9 mm^2 which were designed as shown in Figure S8b. The overall dimension of the mold was $20 \times 20 \times 2\ mm^3$ with quarter-circle (R = 2.5 mm) pillars at the four corners facilitating removal of the PDMS after curing. Each channel was constructed

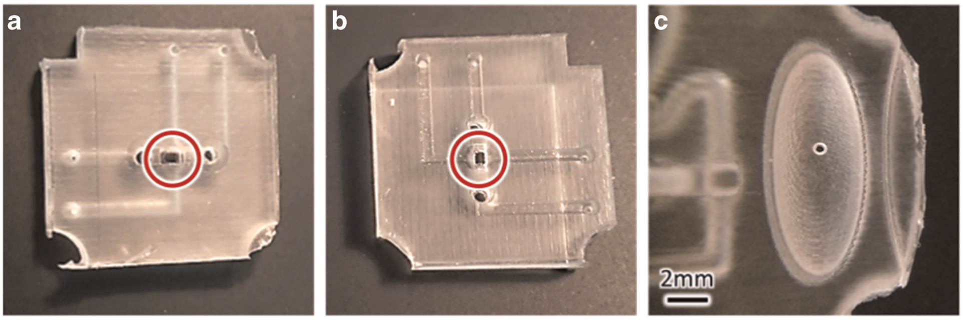

Figure S14 Illustration of membrane damage due to molding problems; points of damage have been circled. "Quake" valve device with ruptured and completely non-formed membrane: **(a)** *Top view* and **(b)** *Bottom view*. **(c)** Elliptical membrane punctured by a bubble-generated 400 μm pinhole.

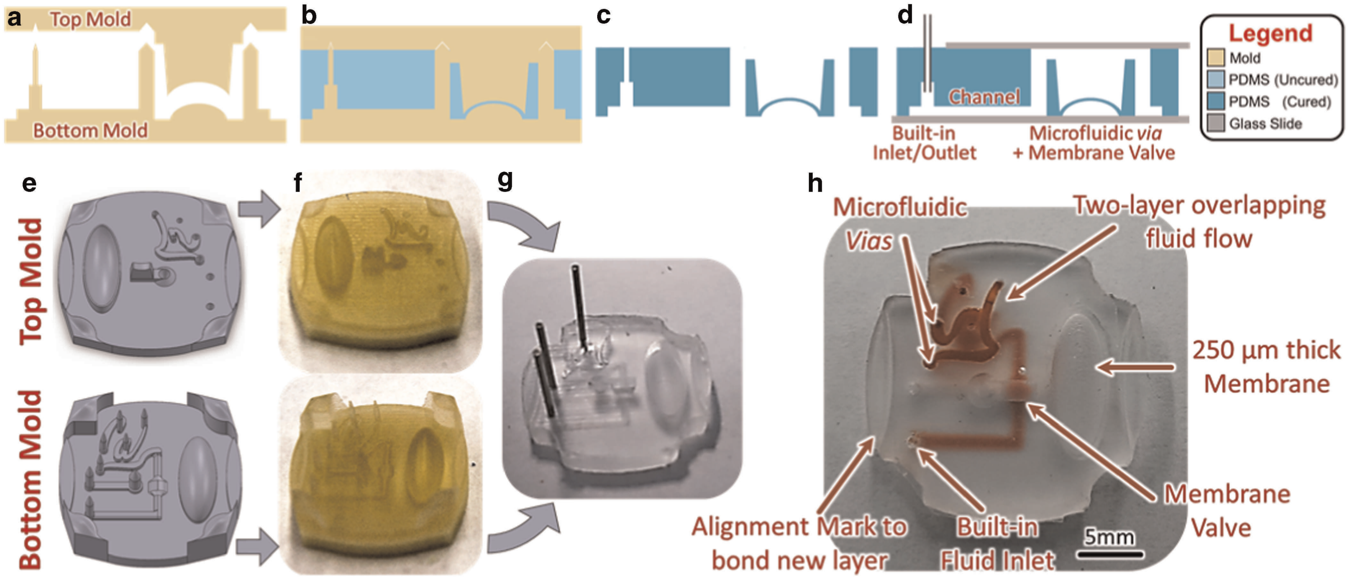

Figure S15 Illustration of the 3D printed transfer molding technique and process for two-sided microfluidic devices with detour vias and thin membranes. **(a)** Mold is 3D-printed from CAD model, treated, fitted together using alignment marks, and **(b)** filled with PDMS which is then cured. Excess PDMS is cut away using a scalpel, after which the mold is removed. **(c)** PDMS device has integrated inlets/outlets, membranes, and vias, and is **(d)** bonded to glass to create enclosed, arbitrary channels. The complex multi-level microfluidic device in **(h)** is fabricated using the CAD models shown in **(e)** and resulting 3D printed molds shown in **(f)**. The devices are then demolded **(g)** and the channels are sealed **(h)** to allow enclosed fluid flow. The final device includes two layers of overlapping fluid flow, an elliptical membrane, a "Quake"-style membrane value, multiple microfluidic vias, integrated fluid inlets, and alignment marks to create a stacked, multi-layer device.

with integrated fluid inlets and outlets molded from pillars 0.55 mm in diameter and 5 mm in height. The inlet pillars were strengthened by widening halfway down their length to 1 mm in diameter. These dimensions are summarized in the schematics shown in Figure S9.

S3.2 Double-sided molding

While the double-sided molding techniques discussed in subsection 'Double-sided channels' are all achieved through the same general process flow, features such as vias and membranes have distinct technical considerations, design rules, and motivations which govern their usage.

S3.2.1 Detour via fabrication. To manufacture microfluidic vias using double-sided molding, the via columns must be designed to fit into the upper mold. In order to prevent PDMS from blocking the top of the via, the designs have the column pierce the upper mold by ≈1 mm and capping it with a conical shape; more intrusion into the upper mold makes the PDMS less likely to fill this space. Similar techniques work for other large-scale features that must be present on both sides of the mold.

Figures S10a and b illustrate conceptual process flows needed to generate a microfluidic overpass vias - used to reroute flow around obstacles such as other channels. First, two molds are printed from CAD file and cleaned and are then fitted together using alignment marks, filled with ℓPDMS, degassed, and baked. Excess PDMS is cut from around molds and PDMS is released and the device is bonded to glass on the top and bottom, taking care to leave input vias uncovered. This technique can be used to fabricate any number of overpass vias, including simple single-pass overpass vias (Figure S10c) and multi- pass crosses with enabled mixing (Figure S10d). Lastly, Figure S10b illustrates fabrication photographs for Celtic Knot-inspired via device including (i) upper and lower molds, (ii) assembled molds, and (iii) the finalized Knot device with interwoven fluid flow and 14 distinct vias.

S3.2.2 Membrane fabrication. Figure S11a illustrates the process flow used to generate arbitrary thin membranes as discussed in subsection 'Thin membranes'. First, a CAD model is designed,

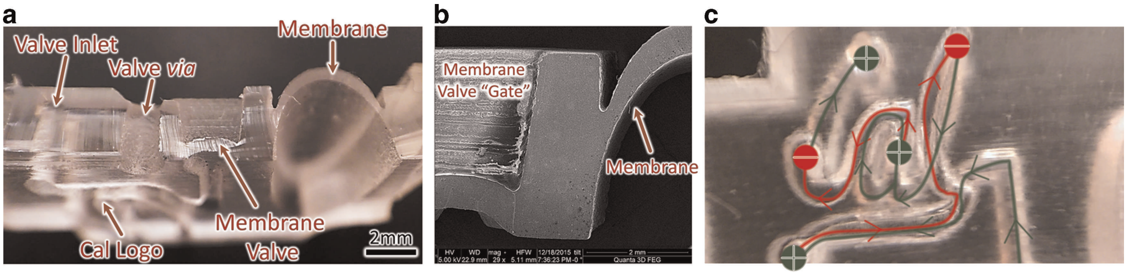

Figure S16 Further explanatory images of the multicomponent microfluidic device from Figure 1. **(a)** Photographic image of membrane cross-section (equivalent to A-A in schematic diagram, but facing opposite direction). **(b)** SEM image of membrane valve and membrane. **(c)** Annotated flow path illustrating the direction of fluid flow through the Cal logo.

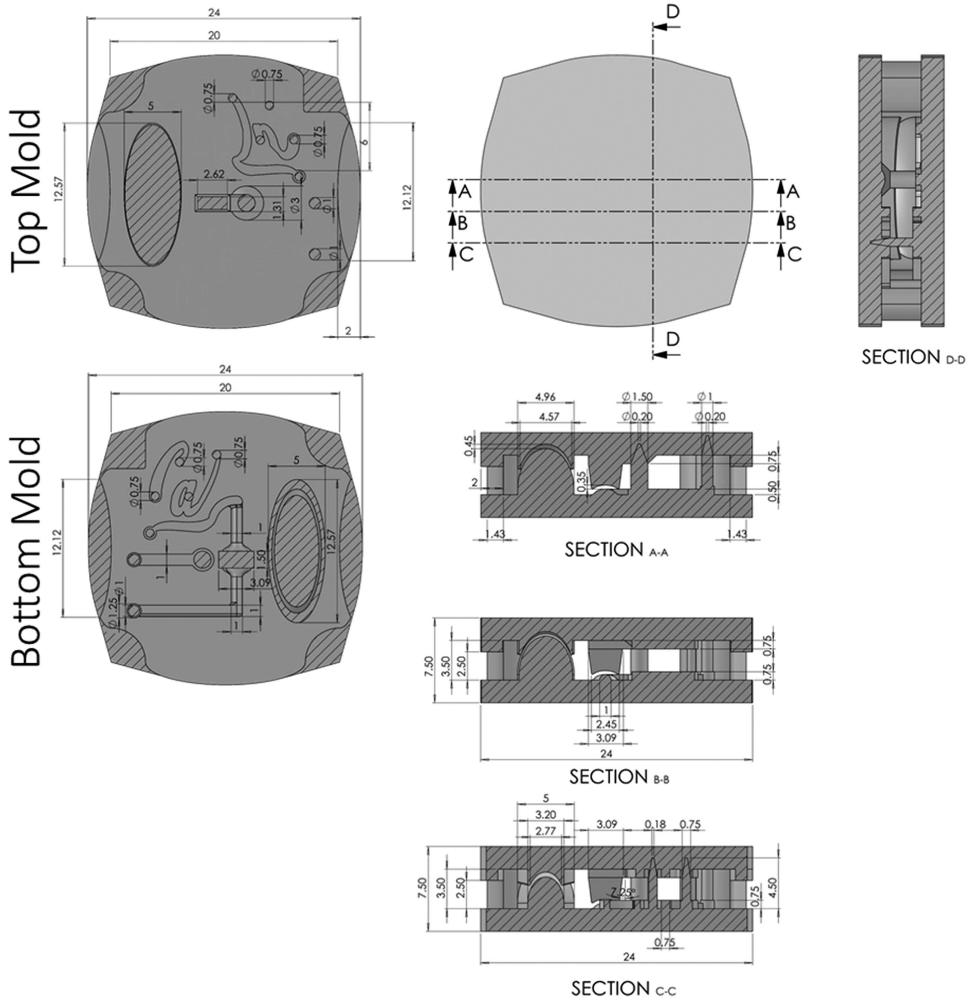

Figure S17 Schematic diagrams for multicomponent microfluidic device molds, including four distinct cross-sections illustrating components perpendicular to the channels.

using the final PDMS design to shape the upper and lower molds; alignment marks and via marks are designed into the mold, ensuring that the membranes nest properly. Next, the molds are filled with ℓPDMS and placed in a vacuum chamber to remove air bubbles from the PDMS, which can rupture the membrane. Note that larger membranes may require longer vacuum times or may require that the molds be vacuum pumped on their side. Finally, the molds are baked to cure the ℓPDMS and demolded. Membranes may be smooth and continuous (Figure S11b) or more irregular (Figure S11c). The sinusoidal pattern in Figure S11c allows for increased membrane deformation and storage capacity without increasing the footprint of the membrane[13]. Schematics of the domed membrane molds are shown in Figure S12. Cross-sectional schematics for the "Quake" membrane valve molds are shown in Figure S13.

Membrane rupture This membrane formation technique can consistently generate membranes down to 200 μm thick, but below that, surface interaction effects interfere with PDMS curing. Because these membranes have not completely cured by the time

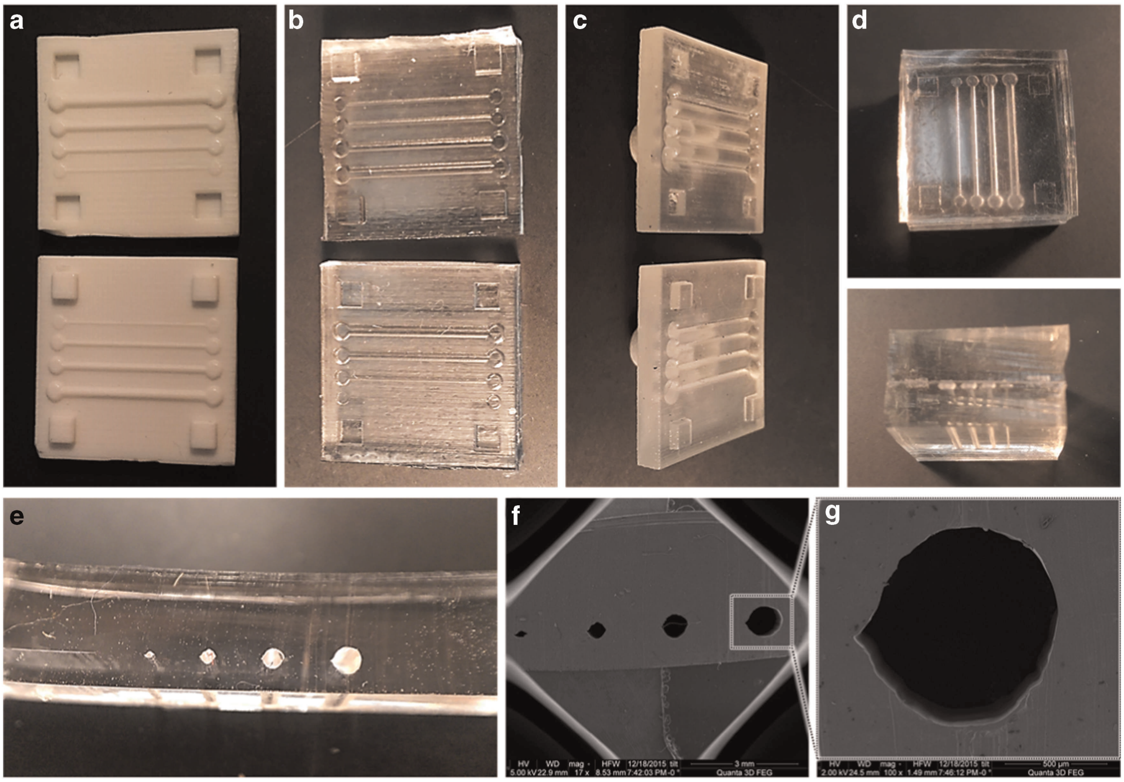

Figure S18 Photographs of process for generating fully rounded microfluidic channels (Radii 500, 375, 250, 125 μm. Images show upper and lower (**a**) 3D printed molds, (**b**) resulting PDMS, and (**c**) corresponding 3D printed stamps. (**d**) Final bonded device with rounded channels seen in top and side view. (**e**) Photographic view of channel cross-sections and (**f**) SEM image of channel cross-section with (**g**) magnification of largest channel, illustrating roundness of channels.

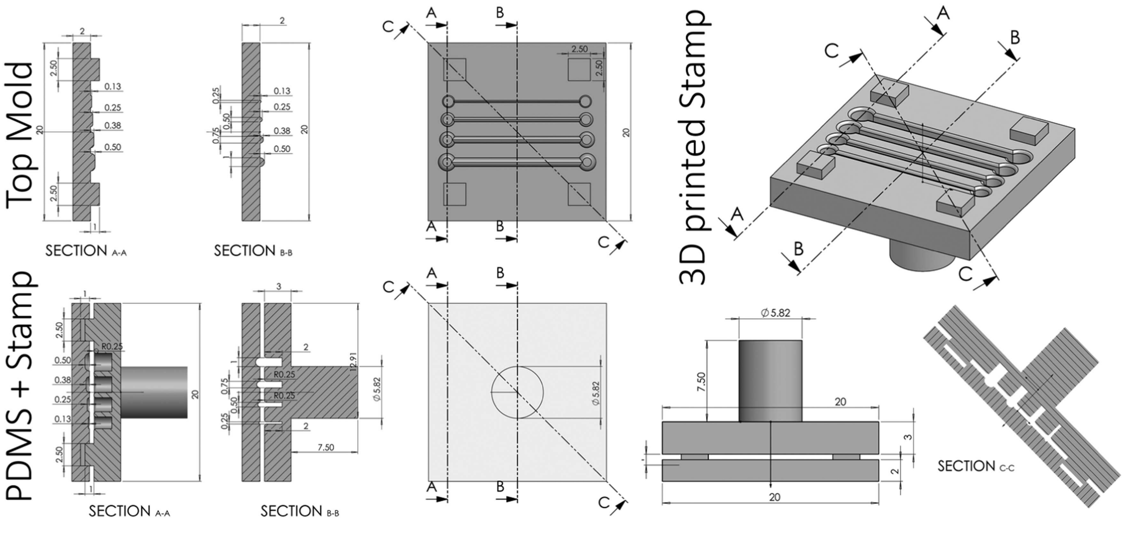

Figure S19 Schematic diagrams illustrating the generation of double sided channels, including the top mold (the bottom mold differs only by intruded alignment marks), the 3D printed stamp, and stamp-PDMS alignment for the upper layer. All cross-sections are located in comparable positions from a top-down perspective.

baking is done, they rupture upon demolding (Figures S14a,b). The exact nature of these effects is not completely known, but is possibly related to lack of oxygen[14]. Additionally, because excessive silanization or liquid absorbed into the 3D printed device interferes with the cross-linking of PDMS, they will also prevent membranes from curing properly. Additionally, if the devices have not spent sufficient time in the vacuum chamber degassing, the presence of bubbles in the membrane will create pinholes upon curing (Figure S14c).

S3.3 Multicomponent microfluidic device

Figure S15 illustrates the process flow for fabricating multi-sided PDMS microfluidic devices using 3D printed molds, expanding on the discussion in section 'RESULTS', with the double-sided molding process shown more clearly (Figures S15e-g). This device has a rounded exterior profile to demonstrate that the device footprint need not be perfectly rectilinear. Note that the Cal Logo is divided up between the upper and lower molds and only shows

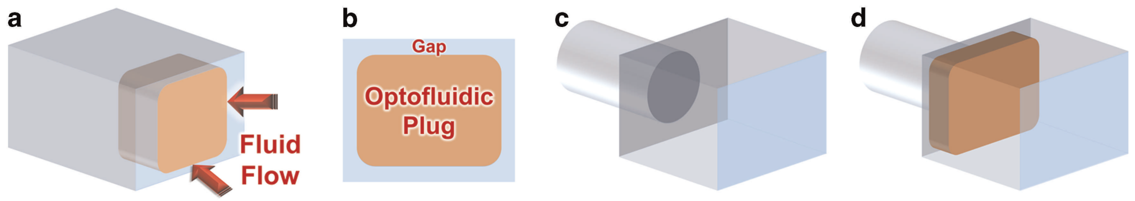

Figure S20 Conceptual diagram illustrating the advantages of PDMS enclosed microfluidic channels (*blue*) with optofluidic hydrogel plugs (*orange*). **(a)** Due to contraction of the optofluidic plug, the plug can move in the microfluidic channel, but **(b)** fluid can leak around the edges of the plug. **(c)** With a variable geometry channel, a plug may be constructed **(d)** to block the smaller portion of the channel but only partially block the larger portion, allowing the plug to both move and not leak.

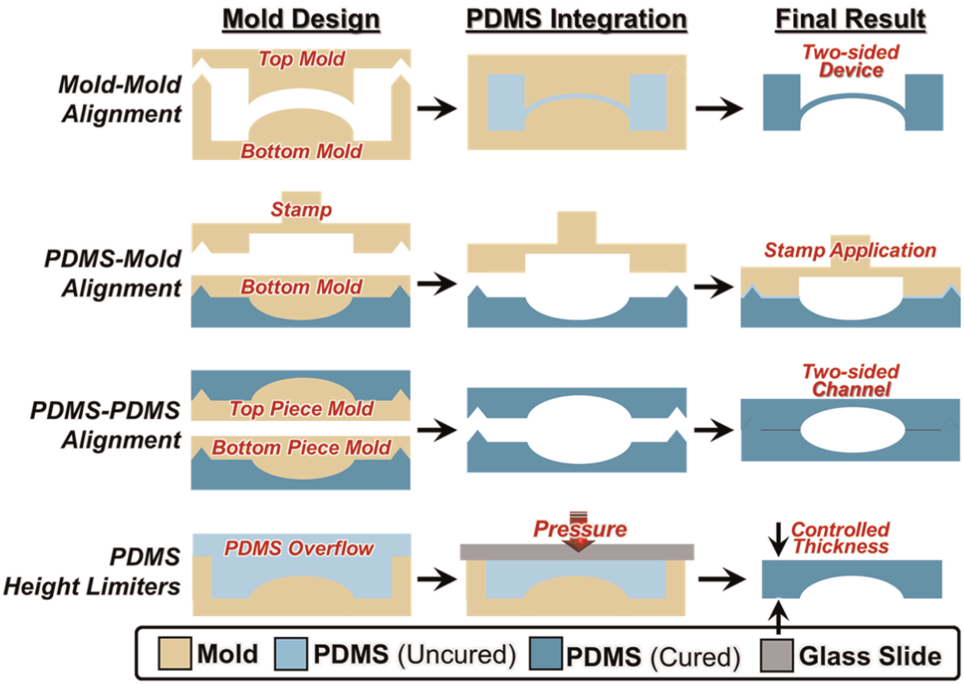

Figure S21 Alignment mark creation process flow. (**a**) Mold-Mold Alignment marks are directly 3D Printed, fitted together, and filled with ℓPDMS. (**b**) Mold-PDMS Alignment marks are directly printed on the 3D stamp and indirectly printed (in negative) on the mold used to generate the PDMS. (**c**) PDMS-PDMS Alignment Marks are both indirectly printed (in negative) onto the 3D printed mold such that the resulting PDMS fits together with the alignment marks. (**d**) PDMS Height-limiters are printed directly onto the 3D printed mold and are flat topped columns onto which a sheet of glass or plastic is placed (under pressure) to keep PDMS to desired thickness.

up clearly in a direct top-down view of the final molded device. Additionally, the mold clearly shows the through-holes for the vias and the integrated inlets and outlets.

Figure S16 shows additional explanatory images of the multi-component microfluidic device. Figure S16a shows a cross-sectional photograph of the membrane valve, with key features labelled. Figure S16b shows the same cross-section, but in SEM image. Figure S16c depicts the flow pattern through the Cal logo, clarifying which at which part of the flow the fluid is in the upper and lower layers, and the direction in which the fluid passes through the intra-layer via marking. Note that the color does not represent fluid flow, but rather the direction of fluid flow. Lastly, schematic diagrams for the device molds are shown in Figure S17.

S3.4 Two sided and fully rounded channels

Currently, there are few techniques for generating rounded channels and each can be quite sensitive. One involves vacuum-loading ℓPDMS into a formed microfluidic channel and then forcing it back out using pressurized air[15,16]. Unfortunately, this technique is unreliable and requires constant attention to prevent blockage of channels by PDMS. Additionally, devices must be specially designed to allow for channel rounding though this technique. Finally, the rounding provided by this technique is only partial (reduction of corner sharpness) and does not lead to truly circular channels. A less method-sensitive technique for rounding rectangular channels uses a positive photoresist (e.g., Shipley SJR 5740) to pattern the fluidic channel molds on a silicon or glass substrate. Then, the substrate is heated to 120 °C for 20 min, heating the photoresist past the glass transition temperature, which allows the resist to reflow and round itself under surface tension[17,18]. Lastly, twofold PDMS replication has been used in consort with CNC milling to fabricate rounded channels, although this technique relies on device symmetry[19].

Figure S18 shows several images of a fully rounded microfluidic channel fabricated using the simpler and more flexible 3D printed transfer molding process, fabricated by bonding two semi-circular channel PDMS devices together. Complementary 3D printed molds (Figure S18a) are used to generate complementary PDMS with integrated PPAM (Figure S18b). Next, the two halves are bonded using ℓPDMS stamp bonding using either stamp pictured

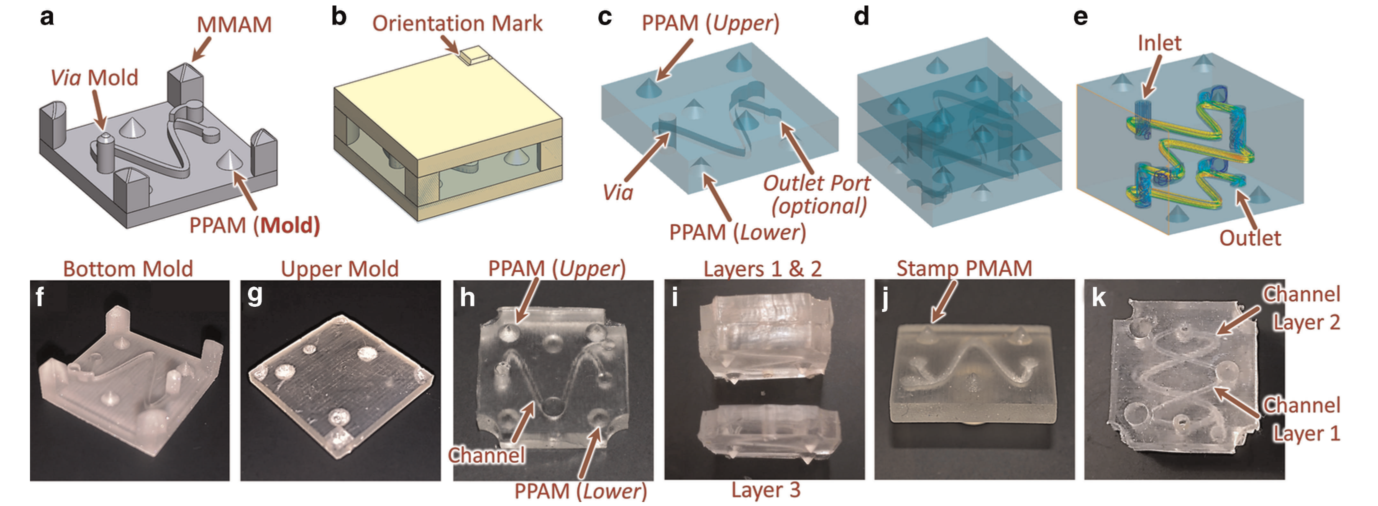

Figure S22 Demonstration of usage of alignment marks. The PDMS-PDMS alignment marks (PPAM) force each layer to reverse 180° relative to the layer below it, allowing for more complicated 3D structures without requiring more molds. *Upper*: Conceptual. *Lower*: Photographs. (**a**) Mold contains pattern for a channel and inter-layer vias, mold-mold alignment marks to align with the upper layer, and conical alignment marks on the bottom and upper surface. The upper surface alignment marks are reversed by 180° relative to the lower surface alignment marks. (**b**) Molds are placed together, with an orientation mark helping ensure that the upper mold is placed in the correct orientation. Note that the structure of the MMAM automatically prevents misalignment, although a visual cue remains helpful. (**c**) Device is demolded after curing process (**d**) Three devices are bonded together (in alternating 180° pattern) via the stamp technique. (**e**) Flow simulation of fluid moving from upper to lower layer. (**f,g**) Device molds and (h) Resulting single layer of cured PDMS. (**i**) Alignment arrangement for bonding third layer and (j) stamp used for ℓPDMS bonding. (**k**) Multi-layered device demonstrating 180° reversal of monomer pattern. relative to the preceding layer.

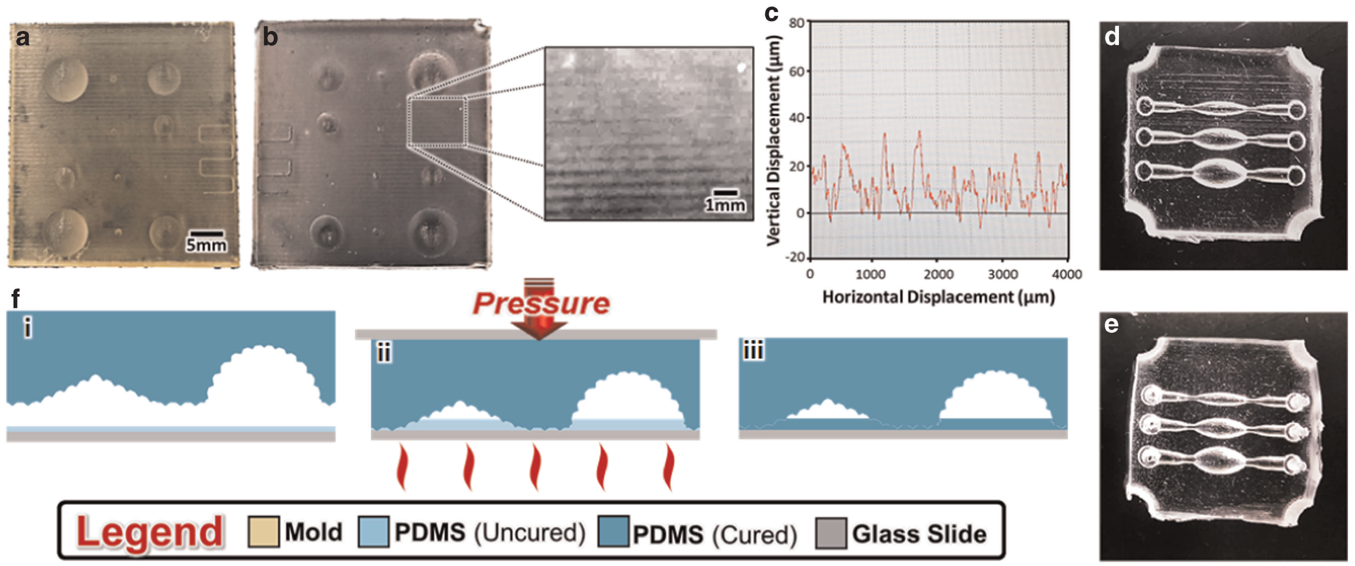

Figure S23 Comparison of results from plasma bonding and ℓPDMS spin bonding. (**a**) 3D printed mold with visible roughness and (**b**) corresponding molded PDMS with magnified insert highlighting roughness. (**c**) Profilometer measurements of 3D printed mold showing $\approx$20-30 μm surface asperity every 0.5 mm. (**d**) PDMS plasma-bonded to glass, showing visible roughness lines. (**e**) Process flow for glass bonding technique. (i) Molded device pressed into spin-coated ℓPDMS. (ii) Pressure is applied while device rests on a 95 °C hotplate. (iii) Cured device. Note that ℓPDMS reflows to fill channels deeper than spin depth. (**f**) ℓPDMS spin bonded device without visible roughness lines.

in Figure S18c. Photographs of completed devices are shown in top-down perspective (Figure S18d) and edge-on (Figure S18e). Figure S18f shows a photograph of a cross section of a device to illustrate the circularity of the channel. SEM images (Figures S18g and h) show that the two halves appear to be a single piece; smooth and mostly circular, with slight kinks where the two halves join together. Schematic diagrams for the double sided channels are shown in Figure S19.

S3.4.1 Uses for two sided and fully rounded channels PDMS-enclosed fluidic channels Creating microfluidic channels which are enclosed on all sides may be useful for microfluidic applications in which fluids or other components cannot be in contact with glass.

(I) Movable components in optofluidic lithography In optofluidic lithography, photocurable hydrogels will permanently bond to a glass substrate[20]. Thus, to fabricate movable components through

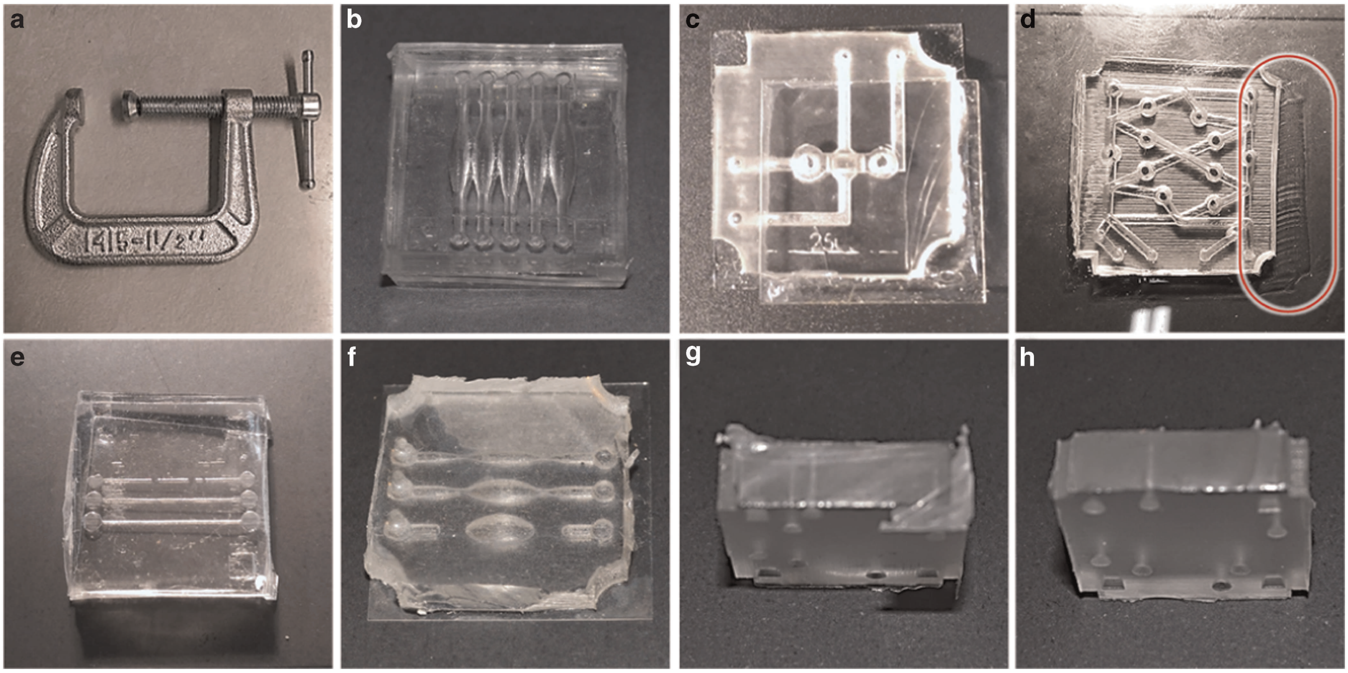

Figure S24 Illustration of problems that occur during glass-bonding. (**a**) 1 ½″ C-clamp used to hold devices in place during bonding (**b**) Device that was excessively torqued during bonding procedure; upper and lower layers do not align properly. (**c**) Device where glass has broken due to thermal expansion during bonding. This is less of an issue in devices which use thicker glass slides. (**d**) Smeared PDMS from lateral motion during ℓPDMS spin-bonding. (**e**) Stamp-bonded device where excess ℓPDMS led to channel clogging. (**f**) Spin-bonded device where a too-thick ℓPDMS layer caused channel clogging. Additionally, due to poor trimming, other parts of the device failed to bond to the glass at all. (**g**) Filaments of PDMS that were not adequately trimmed during de-molding. This is the most common cause of insufficient bonding and may be resolved with a small lip around the edge of the 3D printed mold or by (**h**) trimming the edge bead with a scalpel.

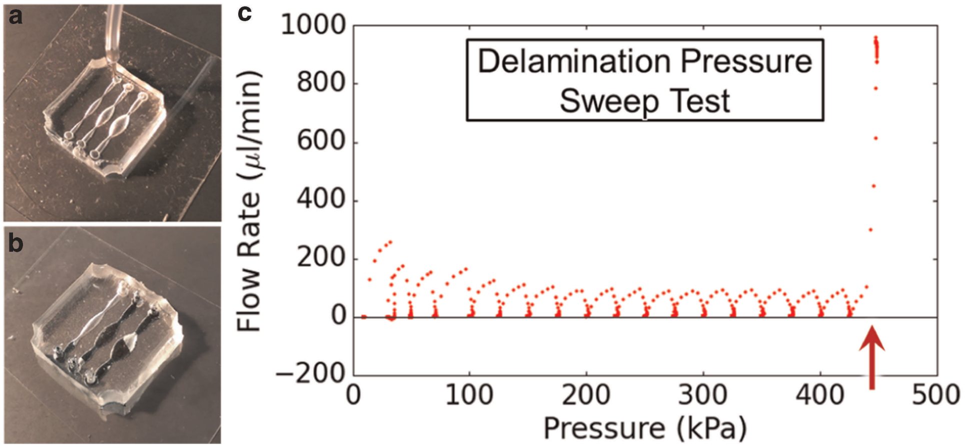

Figure S25 Determination of delamination pressure for glass-bonded single layer microfluidic device. (**a**) Setup for testing of microfluidic device. (**b**) Broken/delaminated device leaking colored fluid between channels (**c**) Graph of results of delamination test; delamination occurred at ≈ 450 kPa. The 'bumps' in the graph are artifacts resulting from the hydraulic capacitance of the system;

optofluidic lithography, the hydrogels must be fabricated within PDMS-enclosed channels. Fabricating microfluidic 'circuit' components using optofluidic lithography requires fabricating channels using standard soft lithography and then bonding to a glass slide that has been spin-coated with a thin layer of PDMS[1,20].

(II) Tight seal against movable components In optofluidic lithography, feature contraction during curing allows components to freely move. However, when used in conjunction with channels with uniform height, this lubricating fluid gap also prevents a tight seal (Figures S20a,b), causing leakage within fluidic diodes or transistor-like components[21,22]. Due to the physics of thin film fluid flow, there is a direct trade-off between speed of optofluidic feature movement under pressure-driven flow and the rate of fluid leakage. For a given uniform thin gap d, pressure drop ΔP and viscosity μ, the leak rate

$$Q_{\text{Leak}} \propto \frac{d^3}{\mu}\Delta P$$

However, the approximate speed of the optofluidic plug

$$V_{\text{Plug}} \propto \frac{d^2}{\mu}\Delta P$$

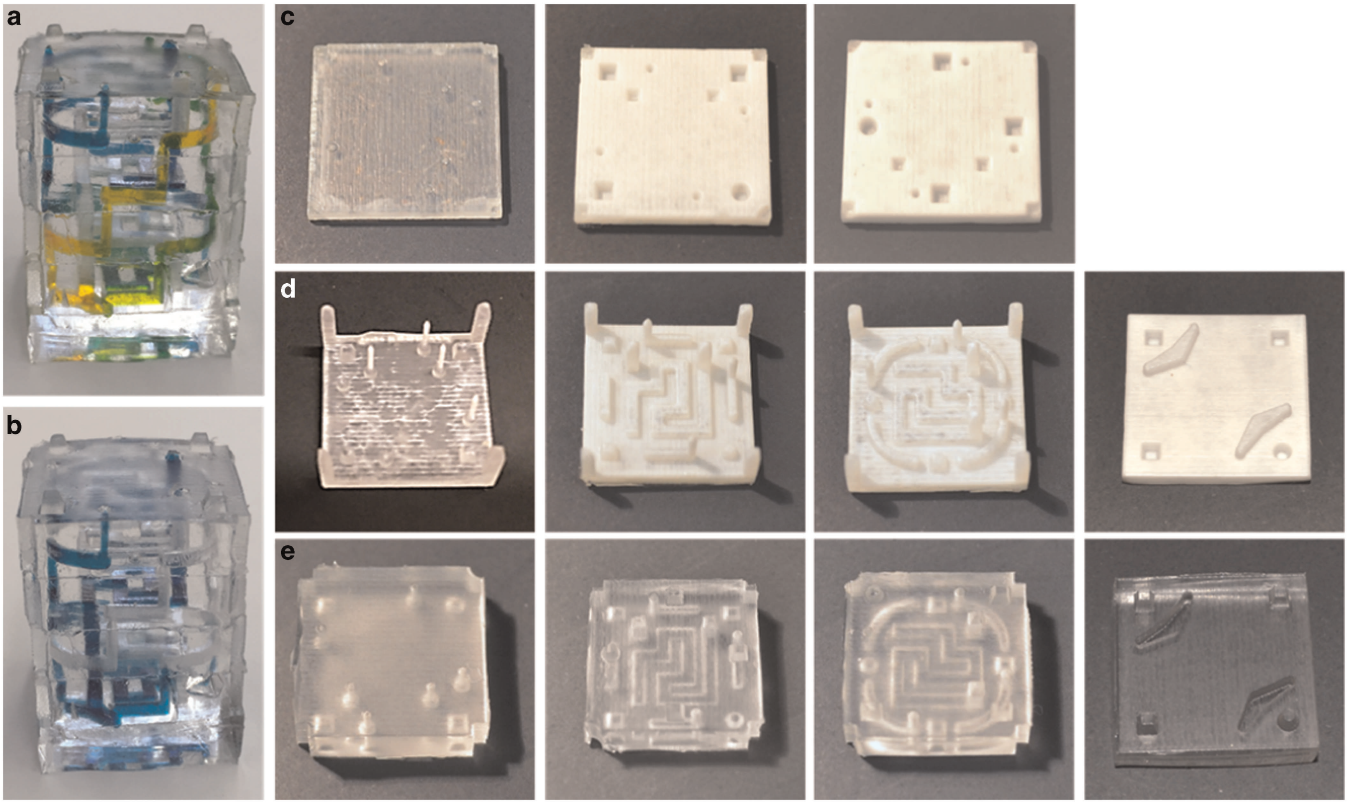

Figure S26 Illustration of Stamp procedure for rapid assembly of multilayer PDMS microfluidic devices achieved with 3D printed molding. *Left*: Photographs of multilayer device with fluid flowing between layers with **(a)** multicolor with fluid mixing, and **(b)** single color. *Right*: layer construction, shown from top layer with integrated inlets (left) to bottom layer (far right). **(c)** Mold upper layers with female alignment marks, **(d)** Mold lower layers with positive alignment marks and microfluidic vias, and **(e)** Resultant PDMS from each layer. Note that the bottom layer only needs a single mold.

While Q_{Leak} scales better with d than V_{Plug}, this scaling still reduces response time to unacceptably slow levels. However, using 3D printed molds, we can fabricate enclosed fluidic channels with non-rectilinear profiles (Figure S20c), allowing optofluidic diodes to move freely in the open position and shut tight in the closed position (Figure S20d). The devices may then be constructed with plenty of movement in the wider channel.

(III) Rounded components for easier membrane valve-sealing Membrane valves do not close effectively when the membrane must fill in channel corners; with rectangular channel geometry, the center of the channel closes first, but the edges of the channels require substantially higher pressures to close. These 'islands of contact' allow fluid to pass by the valve, limiting its effectiveness. However, rounding the upper profile of the channel allows for a smoothing of the actuation-pressure gradient enabling full valve closure at lower actuation pressures. See Unger *et al.* or further discussion[23].

S4ALIGNMENT TECHNIQUES

S4.1 Alignment marks

NB: For the purposes of this discussion, the bottom mold is the mold with the male halves of the alignment marks and the top mold is the mold with the female indentations. The use of 3D-Printed molds enables easy fabrication of PDMS with features molded onto more than one side. While two- sided molding is technically possible for traditional soft-lithography, the process is generally inconvenient due to numerous alignment difficulties. However, using a 3D printer to fabricate the molds, we can build any alignment marks directly into the design, enabling several fabrication techniques. Our research has developed four main varieties of 3D-Printed alignment marks: Mold-Mold alignment marks (MMAM), Mold-PDMS alignment marks (MPAM), PDMS-PDMS alignment marks (PPAM), and PDMS Height Limiters.

S4.1.1 Mold-mold alignment marks Mold-Mold Alignment Marks allow patterns to be imprinted on both sides of PDMS and with desired thickness, with alignment accuracy approximately equal to the resolution of the 3D printer (defects like device warping can lead to greater inaccuracy). These marks consist of a long columnar portion the same height as the thickness of the final PDMS, capped with a male extrusion, extending from the bottom mold, matched a female intrusion (matching the male extrusion) on the top mold. The primary MMAM are generally placed in the corners of the mold, although secondary ones can be scattered throughout the device.

Since MMAM leave vertical holes in the PDMS – the alignment marks move from the bottom to the top of the PDMS – they can be easily adapted to become integrated inputs, vias, and other through-holes in the PDMS. With MMAM narrower than the gauge of a connector syringe, a microfluidic device can be constructed with input-output ports integrated, avoiding the often tedious hole-punching process common to soft-lithography fabrication. Fluidic vias are constructed in a similar manner to smaller Mold-Mold alignment marks, and can be can be constructed that can allow fluid to flow between the top and bottom face of a single layer of PDMS. However, while fluidic vias may be useful in

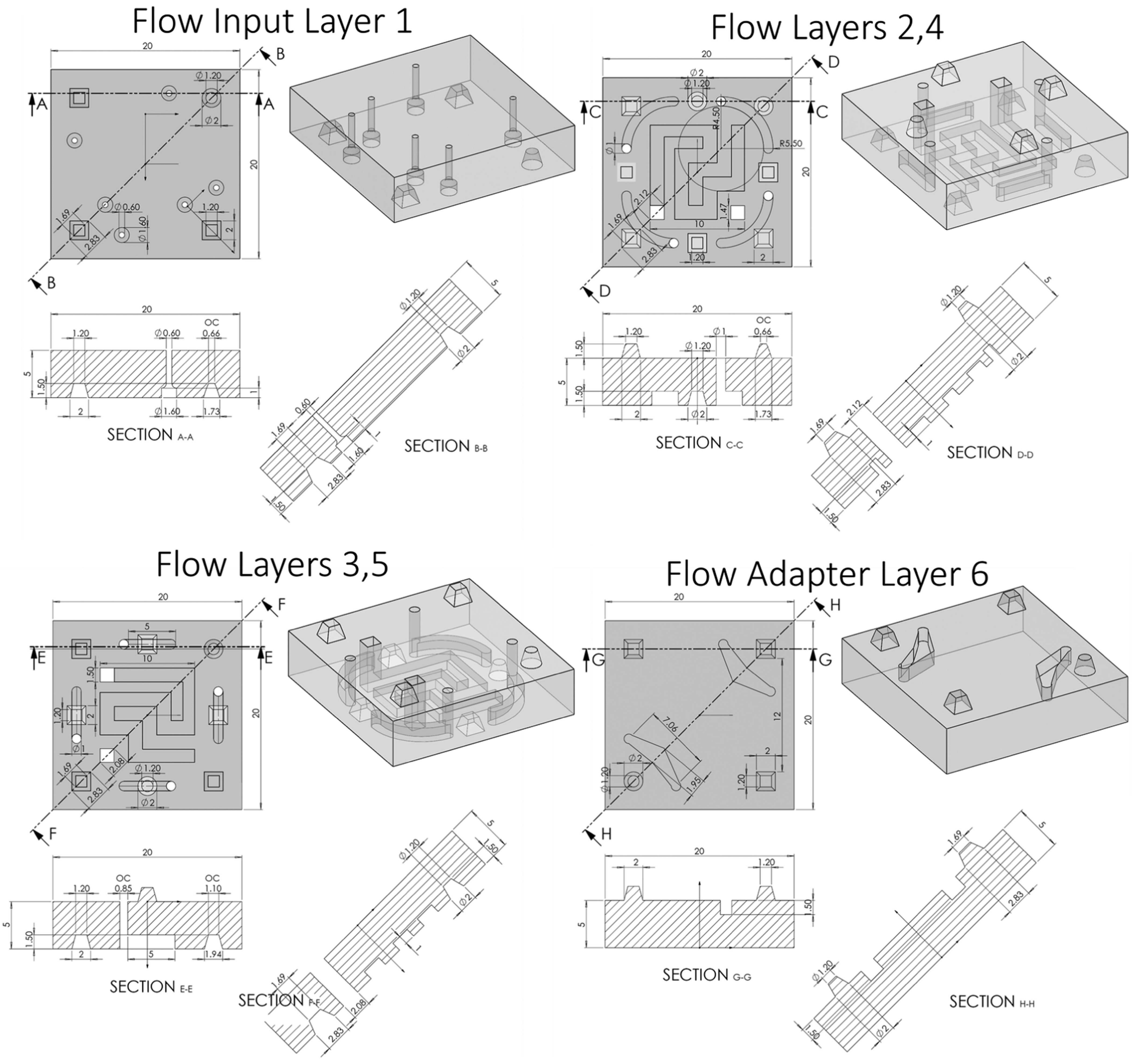

Figure S27 Schematic diagrams for the PDMS layers of multi-layer microfluidic device. The two-sided molds for each component should be reconstructible from the designs for the PDMS layers. Note that non-circular features in the diagonal views do not have the same dimensions as the linear direction. Additionally, *OC* indicates a feature which has dimensions analyzed while the cross-section is off-center.

ensuring a proper rotational orientation of microfluidic devices, using them as MMAM is not recommended due to the narrow gauge and fragility of the via fabrication columns.

S4.1.2 PDMS-PDMS alignment marks PDMS-PDMS Alignment Marks allow multiple layers of PDMS to be easily aligned and bonded. These alignment marks are first built as negatives into the 3D-printed mold and are then transferred to the PDMS. Unlike Mold-Mold Alignment Marks, PPAM generally consist of only the male and female alignment portions (and not the columnar support), since the two PDMS layers must be in full contact for enclosed channels. PPAM may also be constructed on one or both sides of molded PDMS.

A common difficulty with PDMS-PDMS bonding, such as in "Quake" membrane valve technology, is the need to precisely align two or more layers of PDMS. Often, this must be done by hand or using a purpose-built machine to improve accuracy. By building alignment marks directly into the PDMS, a proper alignment can be facilitated (to within the tolerances of the 3D printer) without the need for visual inspection; because the marks only allow the PDMS layers to come into full contact in the correct orientation, alignment can be guided by touch. Additionally, PPAM may be constructed to allow each of the PDMS layers to be aligned in only in desired orientation to fool-proof the device manufacture. Thus, PPAM allow for easy construction of multi-layer PDMS devices.

S4.1.3 Mold-PDMS alignment marks Mold-PDMS alignment marks are a hybrid of the two above methods; they allow a secondary mold (i.e., not the mold that initially imprinted the

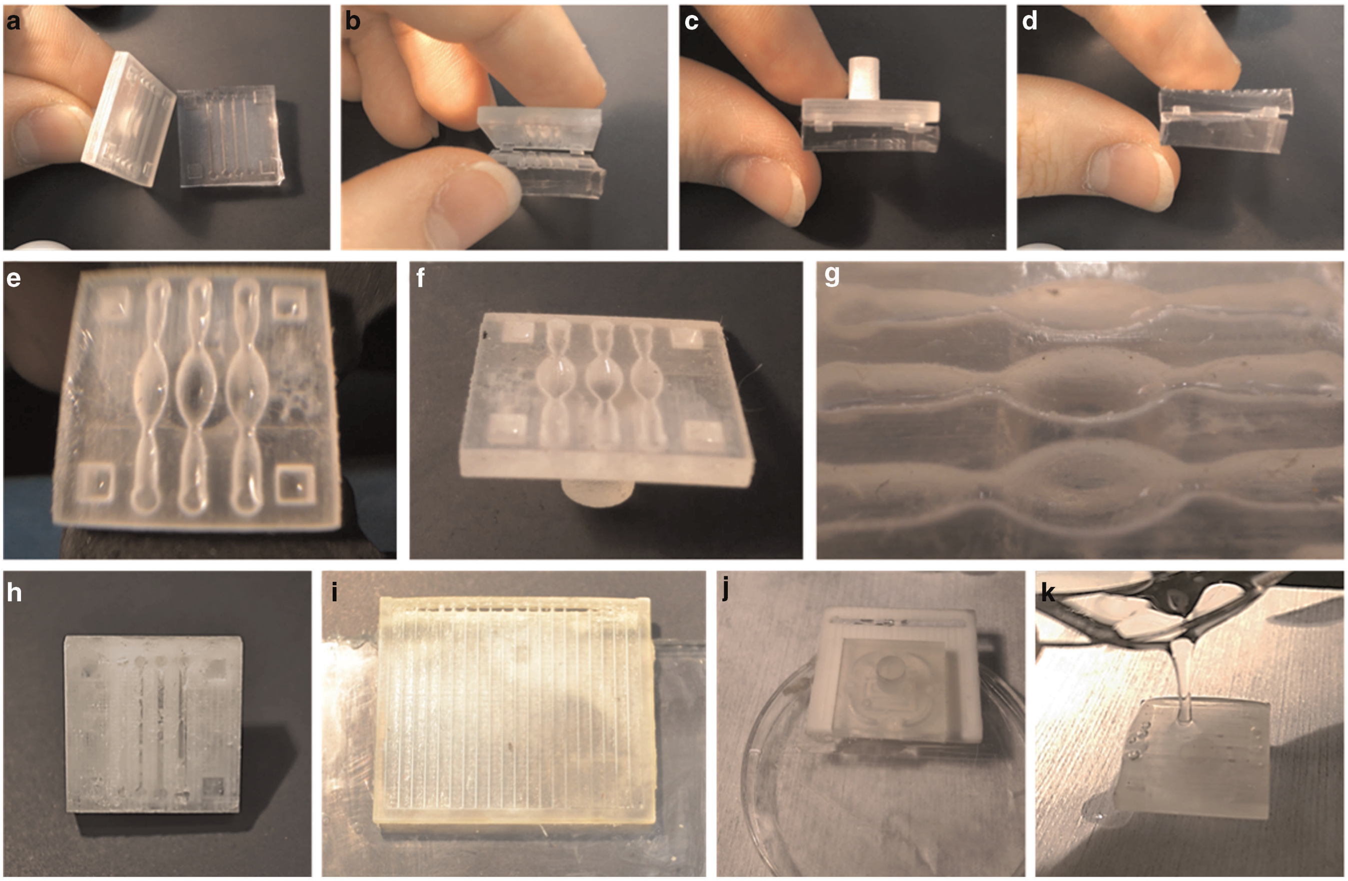

Figure S28 Photographs of stamping technique and other considerations. **(a-d)** Stamping procedure. **(e-g)** Stamp with internally cured PDMS forming curved structure within the channel. The stamp may still be used in this state, but may eventually need to be cleaned with excess ℓPDMS. **(h)** 3D printed ℓPDMS applicator and **(i)** applicator with 3D printed stamp. **(j)** Stamp in need of cleaning with crusted PDMS. **(k)** Stamp cleaning technique: excess ℓPDMS is poured onto the surface, baked, and peeled off as a single unit.

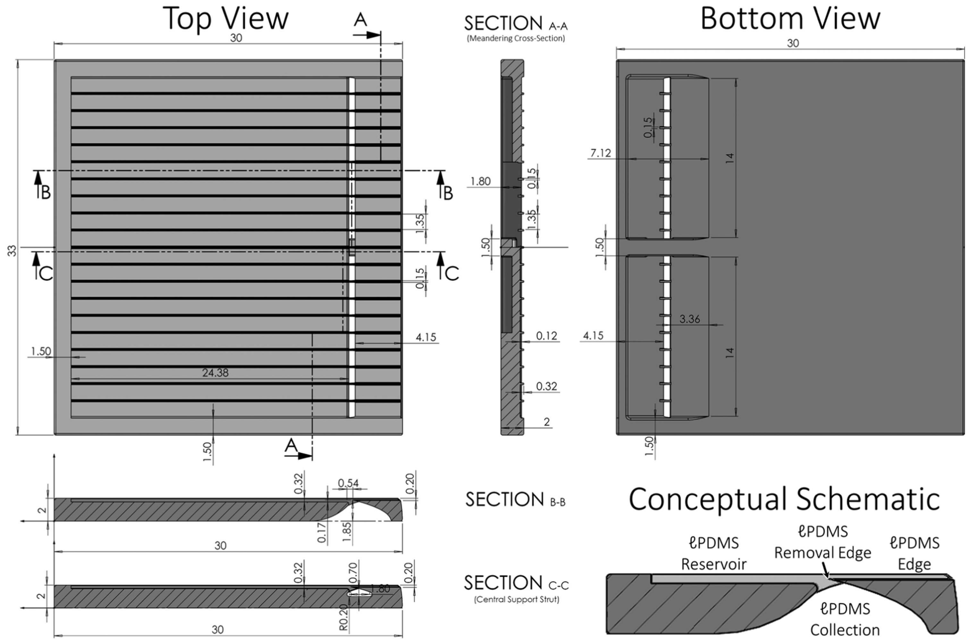

Figure S29 Schematic diagram illustrating the dimensions of the ℓPDMS applicator, including lengthwise cross-sections at the center and towards the edge, a variable cross-section perpendicular to the scraping direction, and a conceptual schematic cross-section.

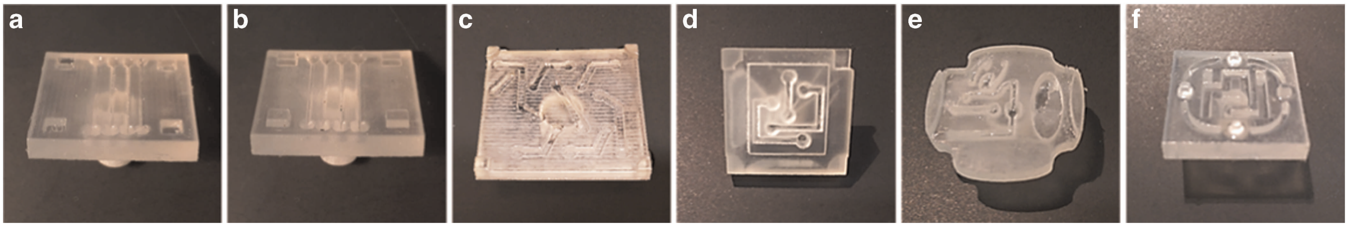

Figure S30 Distinct stamp surface profiles used in multiple experiments. **(a)** Stamp for bonding rounded channel layers (Figure 2i) in intruded and **(b)** intruded stamp topographies. **(c)** Extruded glass-bonding stamp for Celtic 7-knot design (Figure S10). **(d)** Irregular glass-bonding stamp for crossing and overpass device (Figure 2f). **(e)** Glass-bonding stamp for multicomponent device (Figure 1f). **(f)** Extruded PDMS-PDMS bonding stamp for multilayer device (Figure 5).

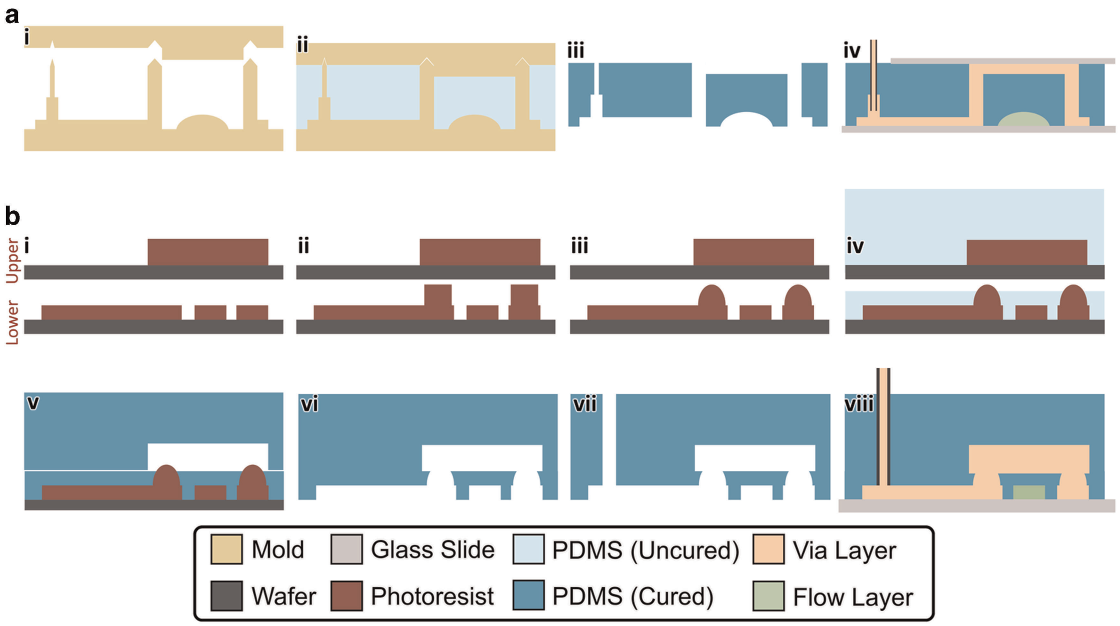

Figure S31 Microfluidic via process flow comparison for (**a**) 3D molding technique, and (**b**) standard soft lithography.

PDMS) to be placed into precise alignment with the PDMS. In order to save space, MPAM are generally designed to fit in the corner cavities left by the primary Mold- Mold Alignment Marks. The PDMS-Mold alignment method is used extensively to align 3D-printed stamps with PDMS.

S4.1.4 PDMS height limiters PDMS Height Limiters are used to keep the thickness of PDMS to a precise level while allowing a smooth PDMS upper surface. Height limiters consist of extruded columns in a single-sided mold with a planar top surface. ℓPDMS is poured onto the mold and degassed, after which a glass (or plastic) slide is placed onto the ℓPDMS and pressed down until it comes into full contact with the Height Limiters. Finally, a weight is placed on the glass slide to maintain full contact during baking.

While height limitation can be accomplished using Mold-Mold alignment marks, the rough surface of the molds tends to leave the PDMS upper surface visually opaque, making a smooth and glass-molder upper surface preferable for single-layer devices. Height limiters are useful in laboratory situations where an oven is not perfectly level, causing later thickness variations, or in situations where a controlled thickness is desired but difficult to achieve. Additionally, due to the individualized nature of the height limiters, multiple PDMS heights (for multiple devices) may be achieved in a single PDMS pour.

S4.2 Alignment mark demonstration

Figure S22 shows a demonstration for the use of alignment marks. This demo is built of stackable identical monomers, and requires two sided molding (and therefore MMAM), and each layer is imprinted with patterns for conical PPAM. The upper mold additionally contains a visual orientation mark (which does not affect the molding) that helps the user identify which corner of the top mold should be oriented toward the unique square (as opposed to quarter-circular) MMAM pillar. The PDMS-PDMS alignment marks on the upper and lower layer of the PDMS are reversed by 180° forcing each layer to be reversed by 180°. Lastly, a flow simulation was run in SolidWorks showing how the fluid progresses from device inlet to outlet (Figure S22e and Video S4.1). The simulation indicates flow streamlines and shows that a small amount of vorticity is imparted to the flow as it moves between layers. Additionally, flow eddies are shown in the optional outlet ports, used to attach syringes in absence of integrated fluid inlets; the port larger to allow hole punching when the device is already bonded.

S5 STRENGTHS AND WEAKNESSES OF BONDING TECHNIQUES

S5.1 Plasma bonding

As in other bonding techniques discussed in this paper, the tightness (but not permanency) of a bonding seal can be judged visually. Non-bonding surfaces appear as areas of incomplete

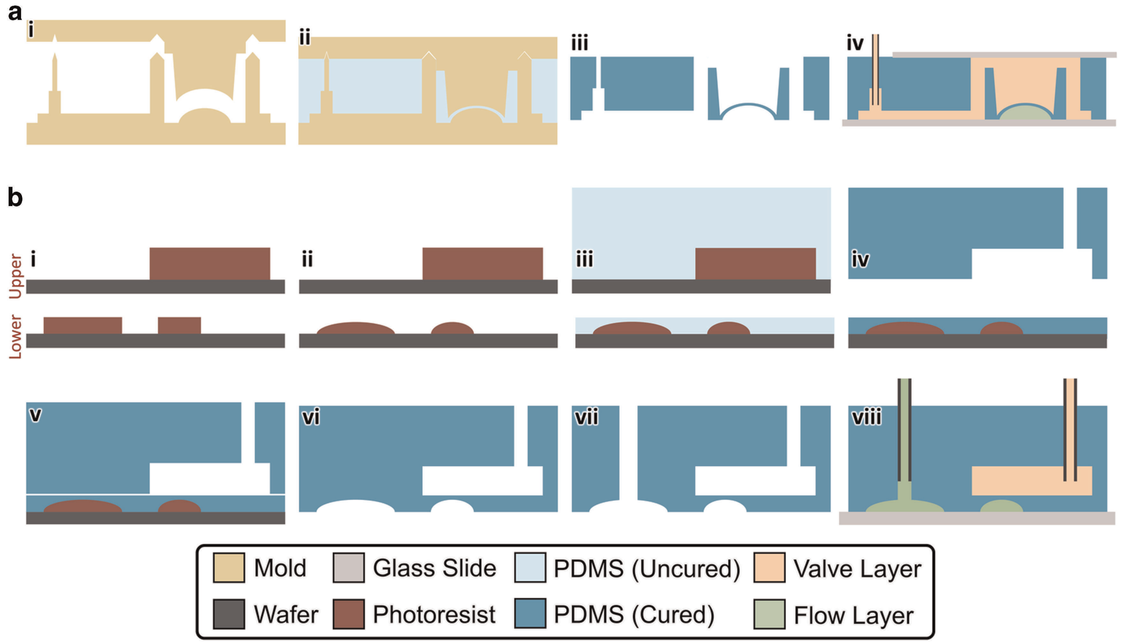

Figure S32 "Quake" valve process flow comparison for (**a**) 3D molding technique, and (**b**) standard soft lithography.

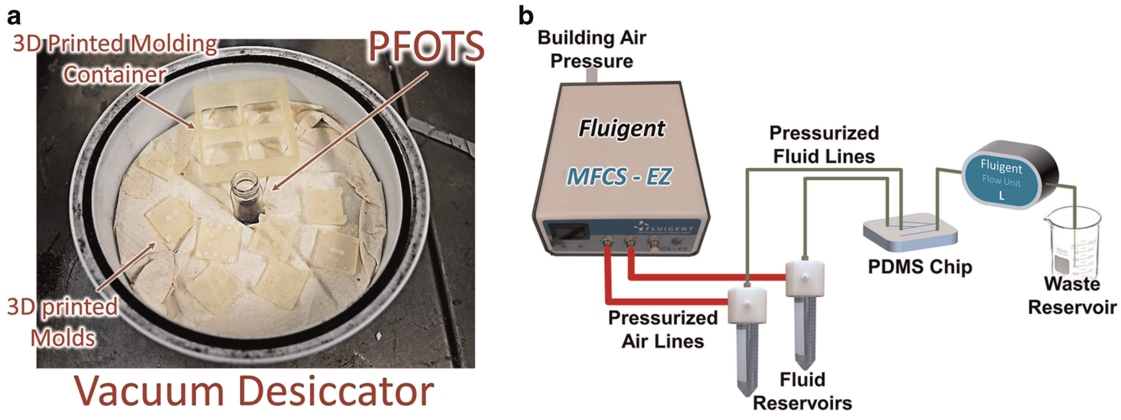

Figure S33 Depictions of the various experimental apparatus used to perform PFOTS silanization and fluid flow rate measurements. (**a**) Bel-Art Nalgene vacuum desiccator for silanization procedure: 1.5 cm glass vial containing 0.3 ml PFOTS is placed in the center among 3D printed components arranged on a paper towel. (**b**) Conceptual diagram showing arrangement of MFCS-EZ pressure pump, PDMS device, and flow sensor.

transparency, white patches, or patches exhibiting interference patterns. When attempting to perform glass-bonding using a standard oxygen plasma process, the components are plasma treated (Plasmod, Nordson March, Concord, CA, USA) for 60 s, placed together, and then baked for 2 hours. However, due to the roughness, a permanent bond is created with numerous vacancies which allow fluid leakage.

To attempt to create a tighter seal, after plasma treatment, our devices were placed in contact with glass, sandwiched between thicker glass slides and then clamped with a 1 ½ C-clamp and tightened until the vacancies vanished. Thicker glass slides were used to distribute the load of the clamps, and glass was used instead of metal due to the transparency; only with glass slides were we able to visually inspect the tightness of the clamp seal. By the time tightening was sufficient, roughly one-in-three devices suffered breakage of one or both glass layers. Additionally, during baking, the relative thermal expansions of glass, PDMS, and metal clamp led to the remainder of devices cracking by the time the devices were ready to extract from the oven. Finally, the high pressure needed to overcome surface roughness resulted in deformed devices, and the rotating nature of the C-clamp led to torqueing of the PDMS against the glass (a problem exacerbated in PDMS-PDMS bonding, even with alignment marks.

Possible resolutions to the above problems include, but are not limited to: using a non-rotating clamping device would likely eliminate torqueing problems and may allow for more precise control of pressures applied. With a calibrated bonding pressure, the glass bonding sheets could be foregone in favor of sturdier metal load distributors. However, the best solution has proven to be use of a PDMS bonding agent to fill in vacancies between layers to be bonded.

S5.2 Glass bonding

S5.2.1 Bonding considerations There are a number of considerations for precise use of the spin bonding technique (discussed in section 'Single-sided molding techniques'), which are outlined

below. Failure in these considerations may lead to device deformities, which are outlined in Figure S24.

(I) PDMS thickness If the PDMS coating on the glass is too thick, excess PDMS has the tendency to clog features in the PDMS device. Some of these issues can be resolved by using a spin-coated PDMS thickness on the scale of the surface roughness to be removed, but smaller than the smallest *features* in the microfluidic device.

(II) Timing dependence A precise calibration of timing and temperature is critical for this technique to proceed satisfactorily. For example, if the PDMS coating on the glass slide cures too much before bonding, or pressure is not applied evenly to the bonding surfaces, the resulting PDMS bond is too weak to be usable. In contrast, if the PDMS does not cure sufficiently, the device tends slip during application causing buildup of PDMS in the channels and creating blockages. Both these difficulties would be resolved by using an integrated clamping/bonding device compatible with a heating system, as opposed to using hand-pressure. The times and temperatures quoted in section 'Single-sided molding techniques' have proven reliable, however.

(III) Planar surfaces Spin-coated PDMS-glass bonding is limited to PDMS pieces with no features protruding beyond the bonding plane, because protrusions prevent a strong bond between the glass slide and the PDMS piece. Additionally, since spin-coating is most effective on flat, featureless devices, this technique cannot be used to make PDMS to PDMS bonds (and therefore multi-layered microfluidic devices). While the PDMS devices may be placed in the spin-coater, the process unsurprisingly leads to PDMS flowing into channels, leading to irreparable clogs and damage to the devices.

S5.2.2 Delamination and inlet bursting 3D transfer molded devices break in three distinct ways. First, the glass itself can fracture under pressure or mishandling. This is a more dominant issue using 0.1 mm thick glass slides, chosen due to the smaller size and favorable shape ($22 \times 22\ mm^2$) than in standard microscope slides. Second, the glass can delaminate from the PDMS, allowing fluid to flow between the glass and the PDMS device. Delamination may be caused by dust particles on the glass bonding surface, by chemical surface contamination, or by over- curing the PDMS device prior to bonding, which inhibits the formation of cross-links between layers. Glass delamination may be mitigated by using very clean glass slides prior to the application of ℓPDMS and not over-curing the PDMS device prior to bonding[14]. Additionally, in the latter two cases, oxygen plasma treatment may help resolve delamination issues. Lastly, high pressure can force the catheter couple out of the device inlet. Catheter ejection issues may be mitigated by using narrower integrated inlets or larger-gauged catheter couples.

Figure S25 illustrates a test to determine the delamination pressure of a bonded microfluidic device (Figure S25a). The fabrication geometry of this system was different than discussed in section 'Single-sided molding techniques'; in this testing geometry, only one side of each channel had integrated inlets, eliminating the need to block off the outlet channels (Figure S25b). Pressure was fed into the device and gradually increased until the glass-PDMS bond broke and fluid began leaking rapidly (Figure S25c). The graph illustrates the resulting $Q-P$ curve, showing a rapid rise in the flow rate as the delamination occurred. The 'bumps' in the graph are artifacts resulting from the hydraulic capacitance of the system; the increase in pressure from the pressure source caused compression of system air and temporary expansion of tubing, leading to an initial flow rate, which vanished as the capacitance became saturated. Delamination is permanent, and further testing of broken devices merely results in rapid increase in flow rate as the pressure is ramped up.

S5.3 3D printed stamp

This section elaborates on many of the techniques shown in section 'ℓPDMS stamp bonding and multilayer rapid assembly', including an expanded discussion of stamp design, ℓPDMS application, and stamp cleaning.

S5.3.1 3D Printed stamp design Figure S26 expands upon the information in Figure 5, illustrating the construction of the multi-level PDMS device. Figures S26a,b show the multi-layer structure from a different angle than seen in Figure 5, one with only blue fluid and one with multiple fluid colors. The device is constructed of four distinct patterns (Figure S26e): a flow input layer, two spiraling flow layers (which are alternated and rotated, and can be repeated indefinitely), and a bottom adapter layer which allows the flow to move between the outward downward flowing spiral and the interior spiral. The layers are constructed from top (Figure S26c) and bottom (Figure S26d) molds. Three distinct stamps are used to apply PDMS to the bottom of the middle layers and the upper layers, enabling PDMS to be applied to the surface without clogging the channels. Schematics for the four distinct PDMS components of the multi-layer microfluidic device may be found in Figure S27.

(I) Stamp filleting Because PDMS beading on the edges of stamp features can potentially be forced into the channels during stamping, and because PDMS applied right to the edge of the channels can be forced into the device during layer bonding, we have filleted (i.e., rounded) the edges of the stamp's complementary channels to reduce blockage during bonding. A fillet radius of curvature of 100-250 µm is sufficient to prevent channel blockage in most cases.

S5.3.2 Application of PDMS to stamp Figure S28 illustrates some techniques to consider when using the 3D printed stamps. After applying ℓPDMS to a stamp, the stamp can be used for 2-3 stampings before needing to reapply ℓPDMS. Additionally, the following stamp techniques may be used to apply PDMS to a glass slide for PDMS- Glass bonding, and tends to reduce the quantity of PDMS that fills the microchannels. However, because the stamp must be used with freshly prepared ℓPDMS, glass bonding can be challenging due to slippage of the glass against the molded PDMS, lubricated by the low viscosity ℓPDMS. We postulate that curing the molded PDMS with applied ℓPDMS for 1-2 min in an 80 °C oven before bonding to the secondary layer might increase the viscosity of the ℓPDMS enough to prevent slippage, making future bonding easier, however we did not perform these experiments.

When a C-clamp is used to bind layers of PDMS together during bonding, care must be taken to not allow the layers to shear against each other (causing lateral displacement) or to torque against each other (causing rotational displacement). Lateral shear emerges most readily when using PDMS that is not of uniform thickness (e.g., from a tilted oven), creating a lateral stress vector resulting from the normal pressure vector. Torqueing shear emerges due to the C-clamp's twisting motion during tightening and may be resolved by using a dedicated apparatus where the torsion is constrained,[24] or through a vacuum bagging method[25]. Vacuum bagging in particular can be used to apply uniform pressure across a bonding interface and has been shown to be reliable for applying laminates in woodworking[26], controlling compressive stress during composite manufacture[27], and for bonding microfluidic devices[25,28].

(I) Direct blotting method ℓPDMS may also be applied to the 3D printed stamps through a blotting technique. Approximately 10g of PDMS is poured onto a TechniCloth cloth and rubbed into the cloth so there is no pooled material. Then the 3D printed stamp (or molded PDMS secondary stamp) is gently pressed into the cloth to transfer ℓPDMS to the stamp. Finally, the stamp is blotted by pressing gently and singly against a dry (non- PDMS-coated) portion of the TechniCloth to remove excess PDMS. (Note that more than one blotting tends to remove too much ℓPDMS and will make the stamping application fail.) Following blotting, the

stamp is ready to apply ℓPDMS to the devices that will be bonded. When PDMS is blotted from the stamp prior to application, excess PDMS is removed from the stamps' complementary channels as well as from the surface. As a result of PDMS surface tension and PDMS fluid continuity, the PDMS tends to form a curved surface in the channel (Figures S28e-g).

(II) Scraper applicator method Precise application of PDMS to intruded stamps can be enhanced slightly through use of a 3D printed applicator (Figure S28i). The applicator is designed with a relatively deep basin to store PDMS (depth = 350 µm but with lateral raised guides to prevent the stamp from sinking more than 100 µm into the basin. Then the stamp is slid along the guides to the edge of the applicator (Figure S28j), where a sharp edge pulls excess ℓPDMS from the mold to collect in the bottom. In some applicator designs, we included a scraper which helped remove excess PDMS from below the depth of the guide rails. After application, the intruded stamp is blotted on a sheet of TexWipe TechniCloth®. For schematics of the ℓPDMS applicator, see Figure S29.

The applicator may be used indirectly to apply ℓPDMS to stamps with extruded alignment marks using a *secondary stamping technique* where an identical copy of the device to be bonded is used as an ℓPDMS applicator for the stamp. First, ℓPDMS is applied to the copy, which is then blotted on TechniCloth. Then the ℓPDMS is transferred to the stamp by pressing the stamp against the PDMS applicator. Finally, the ℓPDMS is transferred from the stamp to the final piece of molded PDMS. Note that this secondary technique may be performed with a reusable 3D printed stamp, but doing so with molded PDMS is often preferable for its lower fabrication time (new 3D printed molds require post-processing whereas there is frequently excess molded PDMS) and lower cost (each molded PDMS has negligible cost once the mold is manufactured).

S5.3.3 Cleaning stamps We recommend that the 3D printed stamps be cleaned at the end of each use, otherwise crusted PDMS will block the channels and leave the stamp useless (Figure S28j). However, because the optimal technique is simple as disposing of excess ℓPDMS, this process is not arduous. This method for cleaning the stamps (or for cleaning molds or the 3D printed applicator) involves using extra ℓPDMS to clean the surface (Figure S28k). ℓPDMS is poured onto the surface of the stamp such that a pool — held together with surface tension — forms. Then the stamp is placed in the oven, curing the excess ℓPDMS for 40 min until fully cured. After curing, the PDMS can be peeled off the surface as a single unit much as in the standard demolding process. This process is generally easier with thicker PDMS layers, and tends to fail for narrow, deep complementary channels in the 3D printed stamp.

The 3D printed stamps may also be cleaned by blotting as much as possible and then rinsing with room-temperature soapy water with a high degree of agitation (note: hot soapy water will speed curing), or with a household cleaner such as GooGone®. Be aware that GooGone has the tendency to absorb into the mold, causing permanent swelling and surface discoloration and should be rinsed off in hot soapy water as soon as feasible. For these reasons, we prefer the cure-cleaning method to GooGone.

S5.3.4 Stamp surface profile The 3D printed stamps can fit into three distinct categories: intruded, extruded, and irregular. Intruded stamps consist predominantly of a single flat plane with intruded channels corresponding to channels in the PDMS and use female Mold-Polymer alignment marks. Extruded stamps are similar, but include at least one male Mold-Polymer alignment mark or other feature extruded from the surface. For both intruded and extruded stamps, all ℓPDMS is applied to a single plane on the PDMS device, and both stamps topographies may use the scraper applicator method (for direct and secondary application, respectively). Irregular stamps apply ℓPDMS to more than one plane or across a curved surface on the molded PDMS devices. Additionally, irregular stamps generally require direct blotting as the ℓPDMS application method, although for non-curved (but still irregular) surfaces, PDMS may be applied through a series of secondary stamping applications.

S6 STANDARD AND 3D MOLDING TECHNIQUE COMPARISONS

S6.1 Via fabrication

(I) 3D Molding process (i) The two molds are printed from CAD file and cleaned. (ii) The molds are fitted together using alignment marks, filled with ℓPDMS, degassed, and baked. (iii) Excess PDMS is cut from around molds and the PDMS component is released. (iv) The device is bonded to glass on the top and bottom, taking care to leave the integrated inlets uncovered. This process is identical to standard double-sided molding technique.

(II) Standard soft lithography process Note that the pattern for the upper layer must be corrected to account for shrinkage in PDMS curing[18,29]. Correction requires the upper mask to be scaled up relative to the lower mask by 0.5% to 1.5% (i) Photolithography is performed to define the mold for the upper layer (thick) and lower layer (thin). (ii) A second photolithography process is performed for the lower layer to create interconnect points for the vias. (iii) The channels for the lower layer are rounded using a heat reflow treatment. Rounding is achieved through surface tension and reduces the likelihood of PDMS covering the via interconnects. (iv) PDMS is poured onto the upper layer. PDMS is spin-coated onto the lower layer, making sure to calibrate thickness such that the interconnects emerge from the PDMS. The upper and lower layers are baked until cured. (v) The upper layer is removed from the mold, and the upper and lower layers are plasma treated. The upper layer is carefully aligned (using visual alignment marks) with the lower layer, placed in secure contact, and baked at 80 °C for 40-60 min to finalize curing. (vi) The bonded layers are removed from mold and (vii) holes are punched in the bonded device using a manual hole puncher. (viii) The bonded, hole-punched device is plasma treated along with the glass slide. The device and the glass are placed in contact (with care to prevent bubble formation) and then cured at 80 °C for 30 min, followed by an all-night incubation to cure.

Additionally, this bonding technique further requires an off-ratio bonding wherein the control (upper) layer uses a 5:1 Base: Cure-agent ratio and the valve (lower) layer uses a 20:1 Base:Cure-agent ratio. During incubation, base and cure agent diffuse across the bond allowing for a more permanent bond connection. This technique is also sensitive to over-curing. This process is described in detail by Thorsen *et al.*[18].

Note that to allow direct fluidic control of via layer (i.e., though catheter couples) rather than passive flow through the vias, an additional hole punching step must be performed between steps (iv) and (v) to allow the hole to enter only into the via layer.

S6.2 Membrane Valve Fabrication

(I) 3D Molding process First, (i) two molds are printed from CAD file and cleaned. (ii) The molds are fitted together using alignment marks, filled with ℓPDMS, degassed, and baked. (iii) Excess PDMS is cut from around molds and PDMS is released. (iv) Device is bonded to glass on the top and bottom, taking care to leave input vias uncovered. This process is identical to standard double-sided molding technique.

(II) Standard soft lithography process for "Quake" valve generation Note that the pattern for the upper layer must be corrected to account for shrinkage in PDMS curing[29]. Correction requires the upper mask to be scaled up relative to the lower mask by 0.5 to 1.5%. (i) Photolithography is performed to define the mold for

upper valve layer (thick) and lower flow layer (thin). (ii) Channels in the lower layer are rounded using a heat reflow treatment to allow for complete closure of the Quake-valve; rounding is achieved through surface tension reflow during heating. (iii) PDMS is poured onto the upper layer. PDMS is spin-coated onto lower layer, making sure to calibrate thickness such that the interconnects emerge from the PDMS. The upper and lower layers are baked until cured. (iv) The upper layer is removed from the mold and holes are punched using a manual hole puncher to allow for direct pneumatic control in the valve layer. (v) The upper and lower layers are plasma treated. The upper layer is carefully aligned (using visual alignment marks) with the lower layer, placed in contact to remove bubbles, and baked at 80 °C for 40 min to finalize curing. (vi) The bonded layers are removed from the mold and (vii) holes are punched in the bonded device using a manual hole puncher. (viii) The bonded, hole-punched device is plasma treated along with a glass slide. The device and glass are placed in contact (with care to prevent bubble formation), and then cured at 80 °C for 30 min, followed by an all-night incubation to cure.

Note that to enable fluidic vias along with the Quake-valve architecture, an additional lithography and development step must be performed between steps (i) and (ii) to allow for the via layer interconnects. This technique also generally requires off-ratio bonding to bond firmly.

S7 EXPERIMENTAL APPARATUSES AND DEVICE SETUP

Vacuum bubble removal and silanization procedures were performed with a Welch ChemStar 1400N Vacuum Pump (Niles, IL, USA), rated to 10^{-4} torr, with devices placed within a 14 cm nalgene vacuum desiccator (Bel-Art Scienceware®). For silanization, ≈0.2 ml PFOTS was placed in an open 1.5 cm glass vial (OD) in the center of the vacuum chamber with devices resting on a paper towel or TechniCloth in the specialized vacuum chamber (Figure S33a). Using a glass vial exposes a constant surface area of the volatile PFOTS, limiting the sensitivity of the process to volume variations in PFOTS. The chamber was sealed and pumped down for 5 min and then the desiccator was sealed and the vacuum pump turned off for a further 30 min. Finally, the chamber was pumped down for 5 min to remove remaining PFOTS vapor from the chamber. After silanization, the molds were ready to use and the PFOTS was returned to a container with Drierite Desiccant (W. A. Hammond Drierite Co., Xenia, OH USA) for storage. Returning the molds to an 80 °C oven for 30 min may help if the ℓPDMS regularly does not cure sufficiently against the surface of the mold. Sometimes, a single sacrificial PDMS pour may be sufficient to remove excess PFOTS particulates from the surface, allowing subsequent moldings from the same mold to occur without difficulty.

Figure S33b shows a conceptual view of the experimental setup used for flow analysis experiments. Building air pressure is passed to the Fluigent MFCS™-EZ system. Pressure is then sent to fluid-filled wells, which then forces fluid to the chip at the desired pressure. On channels for which a flow-rate measurement device was used (Fluigent FlowUnit L), the measurement system was generally placed between the chip and the outlet to allow the chip's internal resistance and capacitance to buffer transient flow rate spikes that result from pressure increases. Because these system-generated transients are of less interest than the stable flow conditions, we find this trade-off acceptable.

REFERENCES

1 Sochol RD, Sweet E, Glick CC *et al.* 3D-printed microfluidic circuitry via multijet-based additive manufacturing. *Lab on a Chip* 2016; **16**: 668–678.
2 3D Systems.VisiJet® EX 200 Plastic MSDS 2009. http://www.3dsystems.com/products/datafiles/visijet/msds/EX200/24184-S12-00-A_SDS_Reach_English_EX_200.pdf. Accessed on 22 Aug, 2016.
3 3D Systems. VisiJet® EX 200 Plastic Datasheet 2009. http://www.3dsystems.com/products/datafiles/visijet/datasheets/Visijet_EX200_Info_0509.pdf. Accessed on 22 Aug, 2016.
4 3D Systems.VisiJet® S100,S300 Support Material MSDS 2012. http://www.3dsystems.com/products/datafiles/visijet/msds/s100-s300/23126-S02-02-B-MSDS-US-VisiJet-S100-S300.pdf. Accessed on 22 Aug, 2016.
5 3D Systems. ProJet™ HD 3000 Technical Specifications 2009. http://www.3dsystems.com/products/datafiles/projet/ProJet_HD_3000_Brochure_USEN.pdf. Accessed on 22 Aug, 2016.
6 Walczak R, Adamski K. Inkjet 3D printing of microfluidic structures-on the selection of the printer towards printing your own microfluidic chips. *Journal of Micromechanics and Microengineering* 2015; **25**: 085013.
7 Mills DL, Maradudin AA. Surface roughness and the optical properties of a semi-infinite material; the effect of a dielectric overlayer. *Physical Review B* 1975; **12**: 2943–2958.
8 Kr J, Zeman M, Kluth O, Smole F, Topi M. Effect of surface roughness of ZnO:Al films on light scattering in hydrogenated amorphous silicon solar cells. *Thin Solid Films* 2003; **426**: 296–304.
9 Ragheb H, Hancock ER. The modified Beckmann-Kirchhoff scattering theory for rough surface analysis. *Pattern Recognition* 2007; **40**: 2004–2020.
10 Pinel N, Bourlier C, Saillard J. Degree of roughness of rough layers: extensions of the Rayleigh roughness criterion and some applications. *Progress in Electromagnetics Research B* 2010; **19**: 41–63.
11 Whitesides GM, Tang SK. Fluidic optics. *Proceedings of SPIE* 2006; **6329**.
12 Dippenaar DJ, Schreve K. 3D-printed tooling for vacuum-assisted resin transfer moulding. *The International Journal of Advanced Manufacturing Technology* 2013; **64**: 755–767.
13 Hwang Y, Paydar OH, Candler RN. 3D-printed molds for non-planar PDMS microfluidic channels. *Sensors and Actuators A: Physical* 2015; **226**: 137–142.
14 Eddings MA, Johnson MA, Gale BK. Determining the optimal PDMSPDMS bonding technique for microfluidic devices. *Journal of Micromechanics and Microengineering* 2008; **18**: 067001.
15 Abdelgawad Mohamed, Wu Chun, Chien Wei-Yin *et al.* A fast and simple method to fabricate circular microchannels in polydimethylsiloxane (PDMS). *Lab on a chip* 2011; **11**: 545551.
16 Sochol Ryan D, Lu Albert, Lei Jonathan *et al.* Microfluidic bead-based diodes with targeted circular microchannels for low Reynolds number applications. *Lab on a Chip* 2014; **14**: 1585–1594.
17 Whitesides GM, Ostuni E, Takayama S, Jiang X, Ingber DE. Soft lithography in biology and biochemistry. *Annual review of biomedical engineering* 2001; **3**: 335–373.
18 Thorsen T, Maerkl S, Quake S. Microfluidic large-scale integration. *Science* 2002; **298**: 580–584.
19 Wilson Mary E, Kota Nithyanand, Kim YongTae *et al.* Fabrication of circular microfluidic channels by combining mechanical micromilling and soft lithography. *Lab on a Chip* 2011; **11**: 1550–1555.
20 Chung SE, Park W, Shin S, Lee SA, Kwon S. Guided and fluidic self-assembly of microstructures using railed microfluidic channels. *Nature Materials* 2008; **7**: 581–587.
21 Glick C, Sochol Ryan D, Wolf Ki Tae *et al.* Pressure gain in single-layer microfluidics devices via optofluidic lithography. 2013 Transducers & Eurosensors XXVII: The 17th International Conference on Solid-State Sensors, Actuators and Microsystems (TRANSDUCERS & EUROSENSORS XXVII); 16-20 Jun 2013; Barcelona, Spain; 2013: 404–407.
22 Glick CC, Peng S, Chung M *et al.* Single-layer microfluidic current source via optofluidic lithography. 28th IEEE International Conference on Micro Electro Mechanical Systems (MEMS); 18-22 Jan 2015; Estoril, Portugal; 2015: 551–554.
23 Unger M, Chou H, Thorsen T, Scherer A, Quake S. Monolithic microfabricated valves and pumps by multilayer soft lithography. *Science* 2000; **288**: 113–116.
24 Li Xiang, Tak For Yu Zeta, Geraldo Dalton *et al.* Desktop aligner for fabrication of multilayer microfluidic devices. *Review of Scientific Instruments* 2015; **86**: 075008.
25 Cassano CL, Simon AJ, Liu W, Fredrickson C, Hugh Fan Z. Use of vacuum bagging for fabricating thermoplastic microfluidic devices. *Lab on a Chip* 2015; **15**: 62–66.
26 David JR *et al.* Method of conforming an adherent film to a substrate by application of vacuum 2005.
27 Torres M, Collombeta F, Crouzeixa Douchina, L *et al.* Monitoring of the curing process of composite structures by tunnelling junction sensors. *Sensors and Actuators A: Physical* 2015; **235**: 256–264.
28 Chen P-C, Liu Y-M, Chou H-C. An adhesive bonding method with microfabricating micro pillars to prevent clogging in a microchannel. *Journal of Micromechanics and Microengineering* 2016; **26**: 045003.
29 Squires TM, Quake SR. Microfluidics: Fluid physics at the nanoliter scale. *Reviews of Modern Physics* 2005; **77**: 977.
